# From Fundamentals to Engineering Sustainable Triboelectric Materials: A Review of Cellulose Platforms

**DOI:** 10.1002/smll.75882

**Published:** 2026-09-23

**Authors:** Renyun Zhang, Dabo Chen, Jiayong Li, Peihong Wang

**Affiliations:** ^1^ Department of Engineering Mathematics and Science Education Mid Sweden University Sundsvall Sweden; ^2^ Beijing Institute of Nanoenergy and Nanosystems Chinese Academy of Sciences Beijing China; ^3^ College of Electrical and Information Engineering Hunan University Changsha China; ^4^ School of Materials Science and Engineering Anhui University Hefei Anhui China

**Keywords:** cellulose nanofibers, cellulose, nanocelluloses, surface engineering, triboelectric nanogenerators

## Abstract

Cellulose has become one of the most promising sustainable platforms for triboelectric nanogenerators (TENGs), driven by its abundance, mechanical robustness, chemical tunability, and compatibility with large‐scale manufacturing. This paper provides a critical and mechanism‐oriented review of how the molecular structure, surface chemistry, and composition of cellulose materials influence charge generation, retention, and dissipation during triboelectrification. We analyze the triboelectric properties of native, regenerated, and nanocellulose materials, as well as the methods developed to enhance their properties. Particular attention is given to surface functionalization strategies—such as fluorination, amination, and esterification that enhance charge density while mitigating humidity‐induced losses. Moreover, the application of cellulose material‐based TENGs has been reviewed extensively. By integrating fundamental insights with engineering considerations, a forward‐looking roadmap is proposed for high‐performance, recyclable cellulose‐based systems for sustainable energy harvesting and self‐powered sensing applications.

## Graphical abstract

This review examines cellulose as a sustainable platform for triboelectric nanogenerators, linking molecular structure, surface chemistry, and material engineering to charge generation, retention, and dissipation. Native, regenerated, and nanocellulose materials, functionalization strategies, scalable manufacturing, and diverse applications are critically discussed. Key challenges, including humidity, durability, mechanistic understanding, scalability, and life‐cycle considerations, are identified to guide future development.

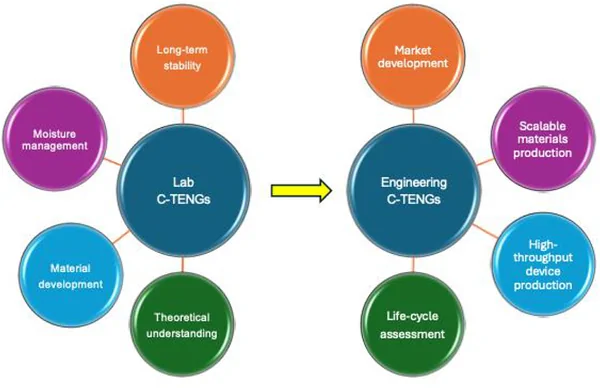

**Abbreviations**
BC: bacterial cellulose
BNC: bacterial nanocellulose
C‐TENG: cellulose material‐based triboelectric nanogenerator
CA: cellulose acetate
CANF: cellulose acetate nanofibers
CFP: cellulose filter paper
CMC: carboxymethyl cellulose
CNC: cellulose nanocrystal
CNF: cellulose nanofiber/cellulose nanofibril
CNN: convolutional neural network
CNT: carbon nanotube
DFT: density functional theory
FEP: fluorinated ethylene propylene
GO: graphene oxide
HMI: human–machine interface
HNG: hybrid nanogenerator
HOMO: highest occupied molecular orbital
IoT: Internet of Things
KPFM: Kelvin probe force microscopy
LCA: life‐cycle assessment
LCD: liquid crystal display
LUMO: lowest unoccupied molecular orbital
MCC: microcrystalline cellulose
MI: methylimidazole
MOF: metal–organic framework
MWCNT: multi‐walled carbon nanotube
NG@P: nanographite‐coated paper
PA: polyamide
PANI: polyaniline
PDA: polydopamine
PDMS: polydimethylsiloxane
PEI: polyethyleneimine
PENG: piezoelectric nanogenerator
PEO: polyethylene oxide
PET: polyethylene terephthalate
PI: polyimide
PLA: polylactic acid
PM: particulate matter
PPy: polypyrrole
PTFE: polytetrafluoroethylene
PU: polyurethane
PVA: polyvinyl alcohol
PVC: polyvinyl chloride
PVDF: polyvinylidene fluoride
PVDF‐HFP: poly(vinylidene fluoride‐*co*‐hexafluoropropylene)
R2R: roll‐to‐roll
RH: relative humidity
SA: sodium alginate
TEMPO: 2,2,6,6‐tetramethylpiperidine‐1‐oxyl
TENG: triboelectric nanogenerator
TEOA: triethanolamine
TEOS: tetraethyl orthosilicate
Ti_3_C_2_T_x_
: titanium carbide MXene with surface terminations
TVB‐N: total volatile basic nitrogen

## 1 Introduction

The need for distributed, sustainable power sources for the Internet of Things (IoT) [1], wearable devices [2], and sensor networks [3] has stimulated the research of triboelectric nanogenerators (TENGs) [4] in the past decade, which convert mechanical motions into electricity through coupled contact electrification and electrostatic induction. High‐output devices relied mostly on fluoropolymers such as polytetrafluoroethylene (PTFE) [5] and fluorinated ethylene propylene (FEP) [6], whose chemical inertness and strong electron affinity favored large charge transfer during triboelectrification. However, the environmental footprint, limited recyclability, and end‐of‐life challenges of such polymers motivate a transition toward renewable, biodegradable dielectrics. Cellulose—the most abundant biopolymer on Earth—offers an excellent alternative. It combines mechanical robustness, low density, and a rich surface chemistry that is readily modified to tailor polarity [7], dielectric response [8], and interfacial adhesion [9] to enhance triboelectric performance.

Over the past few years, cellulose‐, paper‐, modified or composite cellulose‐based TENGs (C‐TENGs; we use this abbreviation generally for all cellulose material‐involved TENGs) [10, 11, 12, 13, 14, 15] have progressed from proof‐of‐concept demonstrations to engineered platforms that exhibit power densities comparable to those of engineering polymers (Figure 1). Reports of fully green TENGs that achieve an output of ∼300 W/m^2^ [15], regenerated cellulose films with solvent‐controlled polymorphs [16, 17] and tribo‐polarity [18], and scalable triboelectric papers [19] fabricated using papermaking‐compatible processes collectively illustrate the great potential of cellulose materials. In addition to the inherent sustainability, cellulose integrates naturally with existing paper infrastructures. These make cellulose an excellent candidate for achieving industrial‐scale production of TENG‐based devices, such as environmental sensors, smart packaging, and large‐area energy interfaces.

**FIGURE 1:**
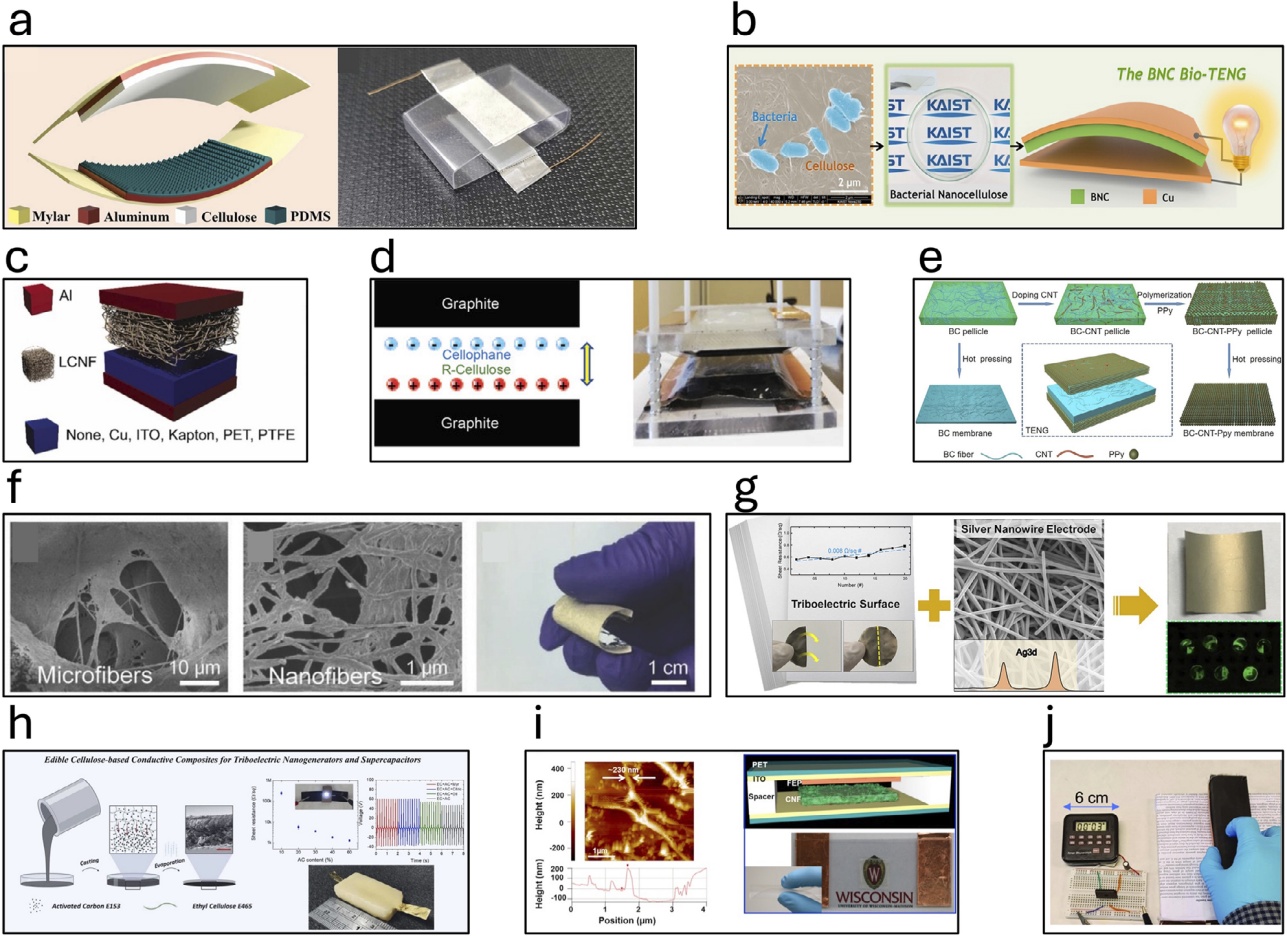
Cellulose materials‐based TENGs. (a) Cellulose acetate nanofiber‐based TENG. Reused with permission from ref. [20]. Copyright 2022, Elsevier. (b) Bacteria Nano‐cellulose TENG. Reused with permission from ref. [21]. Copyright 2017, Elsevier. (c) Natural lignocellulosic nanofibrils‐based TENG. Reused with permission from ref. [22]. Copyright 2022, Elsevier. (d) Regenerated cellulose‐based TENG. Reused from ref. [15] under CC BY 4.0. (e) Recyclable bacteria‐cellulose TENG. Reused with permission from ref. [23]. Copyright 2021, Elsevier. (f) Nanostructured nanocellulose fiber‐based TENG. Reused with permission from ref. [24]. Copyright 2018, Wiley. (g) Cellulose nanofiber‐based TENG. Reused with permission from ref. [25]. Copyright 2018, Elsevier. (h) Edible cellulose TENG. Reused from ref. [26] under CC BY 4.0. (i) Renewable cellulose nanofibril‐based TENG. Reused with permission from ref. [27]. Copyright 2023, Elsevier. (j) Wastepaper‐based TENG. Reused from ref. [28] under CC BY 4.0.

Several previous reviews have summarized cellulose‐based piezoelectric and triboelectric nanogenerators, lignocellulosic feedstocks, material‐modification strategies, wearable devices, and the general opportunities of cellulose in triboelectric systems. The present review does not seek novelty through a catalogue of additional device demonstrations. Instead, it establishes an integrated mechanism‐to‐manufacturing framework. This review focuses on the fundamentals of the triboelectricity of cellulose, the fabrication of cellulose triboelectric films, the engineering of cellulose‐based triboelectric materials, and the challenges to be addressed in the near future. We first introduce the physics of contact electrification at cellulose interfaces and discuss charge generation, retention, and dissipation. We then explore materials design—from native and regenerated cellulose to nanocellulose composites and chemically modified papers—and connect these chemistries to the performances of TENGs. Besides, the applications of TENGs have been reviewed. Subsequently, we discuss scalable fabrication and robustness strategies, followed by a discussion of future challenges. Finally, we outline a roadmap for forward‐looking circular, multifunctional C‑TENGs.

## 2 Fundamentals of Triboelectrification in Cellulose Materials

### 2.1 Contact Electrification and Electrostatic Induction

TENG operation involves two tightly coupled processes [29]: (i) contact electrification during approach and intimate contact of two surfaces, and (ii) electrostatic induction when the surfaces separate, driving electrons in the external circuit to balance the evolving potential difference. In cellulose‐based material pairs, contact electrification can proceed through electron transfer [30], ion transfer mediated by adsorbed water or residual salts [31], and field‐induced dipole polarization [32]. Conditions such as humidity, counter material, surface states, and mechanical load can significantly influence contact electrification, and recent work has highlighted the important role of interface mechanochemistry in the magnitude, polarity, and reproducibility of triboelectric charging [33].

### 2.2 Molecular Origins of Charge Transfer at Cellulose Interfaces

Cellulose‐based materials are placed at different places in the triboelectric series because the modification of the cellulose molecule can significantly change its triboelectric properties. Moreover, the crystallinity of cellulose, arising from the interplay of hydroxyl groups, inter‐ and intra‐chain hydrogen bonds, and local surface states, can also affect the triboelectric effect. Hydroxyl orientation and density, as well as crystalline allomorphs (e.g., cellulose I and II), and surface reconstruction, modulate electron affinity and the depth of trap states [21, 22, 23, 24]. Density functional theory (DFT) (Figure 2a) and molecular simulation (Figure 2b) indicate that the highest occupied molecular orbital (HOMO) and the lowest unoccupied molecular orbital (LUMO) of cellulose are close to those of nylon [34], which suggests a positive charge affinity of pristine cellulose. Experimental results have confirmed these theoretical predictions, showing that the triboelectric charge density of regenerated cellulose (cellulose II) is among the strongest positive‐charged affinity polymers (Figure 2c) [15]. Reports have also shown that the introduction of electron‐withdrawing groups (e.g., ─CF_3_, ─NO_2_) can increase the surface electronegativity and stabilize negative charge (Figure 2d) [35], whereas amination or quaternization enhances donor behavior [36]. Moreover, nanoscale roughness and porosity have been found to increase the active contact area and local field concentration, thereby amplifying charge separation [27].

**FIGURE 2:**
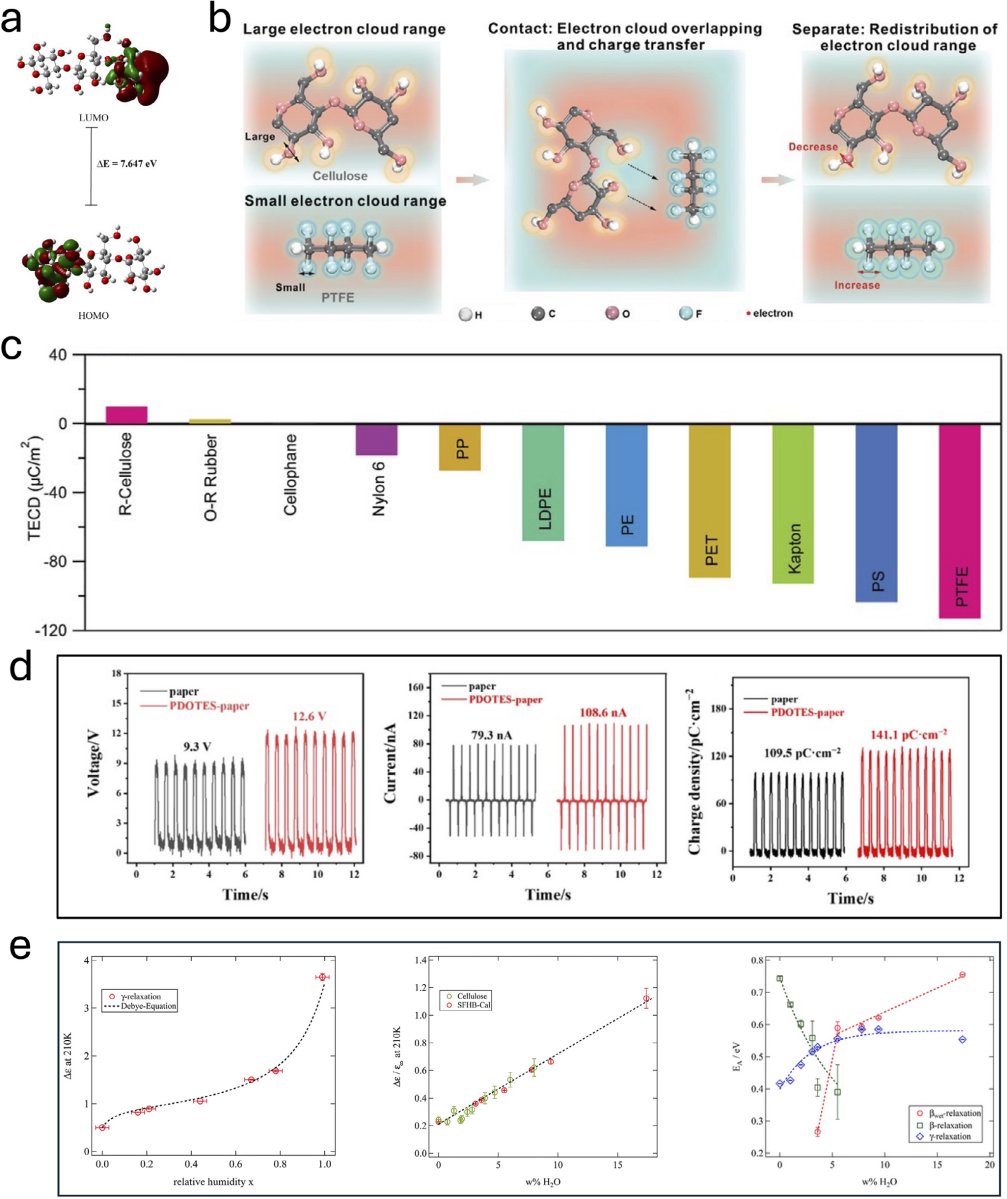
The triboelectric properties of cellulose. (a) The HOMO and LUMO orbitals of cellulose. Reused from ref. [37] under CC BY 4.0. (b) Schematic diagram of cellulose and PTFE contact electrification. Reused with permission from ref. [38]. Copyright 2022, Wiley. (c) The triboelectric charge density of regenerated cellulose compared to other polymers. Reused from ref. [15] under CC BY 4.0. (d) The output of the PDOTES‐paper‐based TENG. Reused from ref. [35] under CC BY 4.0. (e) Relaxation strength *Δε* of the γ‐relaxation at 210 K against relative humidity during previous sample storage, normalized relaxation strength of the γ‐relaxation at 210 K plotted against the mass fraction of water present in the sample, and Results of fitting an Arrhenius function to the mean relaxation times of the SFHB‐Cal sample depending on the water fraction in the sample. Reused from ref. [39] under CC BY 3.0.

### 2.3 Charge Retention, Humidity, and Dielectric Relaxation

Unlike highly hydrophobic fluoropolymers, cellulose is a hydrophilic material because of the abundance of hydroxyl groups within its structure. This natural hydrophilicity means that cellulose‐based materials are inherently sensitive to environmental humidity, a significant challenge for their use in TENGs [40, 41].

Water adsorption is a key factor that significantly affects the electrostatic properties of cellulose in TENG applications. When moisture is adsorbed, it is the surface charge that increases surface conductivity [42, 43, 44], which can rapidly accelerate charge dissipation, especially at relative humidity (Figure 2e) levels above 50%–60% (water uptake transitions and secondary adsorption [39, 45]). Dielectric relaxation processes in hydrated cellulose result in a time‐dependent potential decay [39, 46], compromising the stability and consistency of the TENG output. Quantitatively, the effective surface charge density (σ_*eff*_) inferred from the open‐circuit voltage (*V_OC_*) and electrode geometry often decreases significantly with increasing humidity [47, 48]. The slope of this decrease is dependent on material characteristics such as surface energy and porosity.

On the other hand, the influence of humidity on C‐TENGs depends on the structure of the films made from cellulose materials. Usually, cellulose fiber–based films are percolated, allowing moisture to penetrate and bind to the fibers. Such a binding significantly reduces the charge affinity during the contact‐separation processes. Percolation in the cellulose film could be one of the reasons for moisture penetration. A high‐density, low‐percolation cellulose film would obviously reduce the impact of moisture. We have demonstrated that a high‐density film of regenerated cellulose can prevent the penetration of humidity, thereby stabilizing the output of a TENG over the tested humidity range of 30%–84% [15].

## 3 Triboelectric Cellulose Materials

### 3.1 Native and Fibrous Cellulose Platforms

The fundamental distinction between native and regenerated cellulose lies in their structural differences and origins. Native cellulose is the pristine polymer found in plant cell walls, possessing a highly ordered crystalline structure. In contrast, regenerated cellulose [49] is a man‐made material produced by dissolving native cellulose using a solvent and then regenerating it. This process permanently alters the internal architecture, resulting in a material with different crystallinity, physical properties, and appearance.

Native cellulose is the cellulose I allomorph that exhibits a semi‐crystalline morphology stabilized by an extensive intra‐ and intermolecular hydrogen‐bonding network. For regenerated cellulose, the crystalline structure undergoes an irreversible polymorphic transition to the more thermodynamically stable cellulose II allomorphs [50]. This conversion changes the chain packing and polarity, replacing the parallel chain alignment of cellulose I with an antiparallel orientation in cellulose II [51]. For applications in advanced TENGs, this structural shift is critical, as it can enhance charge capture, which affects triboelectric output. Furthermore, solvent‐engineered regeneration allows precise control over the film's porosity, roughness, and dielectric properties, enabling the fabrication of high‐performance, flexible TENGs that surpass the capabilities of their native cellulose counterparts [15].

### 3.2 Regenerated Cellulose

Recent studies have demonstrated that solvent‐engineered cellulose films (Figure 3a) provide a powerful strategy to actively change cellulose's position along the triboelectric series, enabling its rational pairing with counter‐tribolayers for maximum Δ*χ* (triboaffinity difference) and enhanced power output [17, 41, 52]. The underlying mechanism hinges on the precise control that different solvent systems—such as ionic liquids, alkali/urea aqueous solutions, or deep eutectic solvents—exert over the supramolecular structure and surface chemistry of the regenerated cellulose. For instance, research by Yang et al. showed that specific solvent systems can yield films with a higher density of exposed polar hydroxyl groups, making the surface more tribo‐negative. In contrast, others can promote a different crystalline packing or introduce non‐polar groups that moderately decrease its electron‐withdrawing strength [17]. This tunability enables cellulose to be strategically positioned from a strongly positive material to an intermediate one, and to be paired with nylon and polyvinyl alcohol (PVA) [43]. Furthermore, this solvent‐mediated engineering has been shown to alter other essential properties, including surface smoothness for greater contact intimacy and the dielectric constant for more effective charge induction, thereby synergistically boosting the TENG's performance. The emerging paradigm, as highlighted in forward‐looking research, is the creation of “triboelectric paper” in which the regeneration process is finely tuned not only for mechanical flexibility but also for targeted triboelectric functionality, opening new avenues for sustainable energy harvesting and intelligent, self‐powered sensing [52].

**FIGURE 3:**
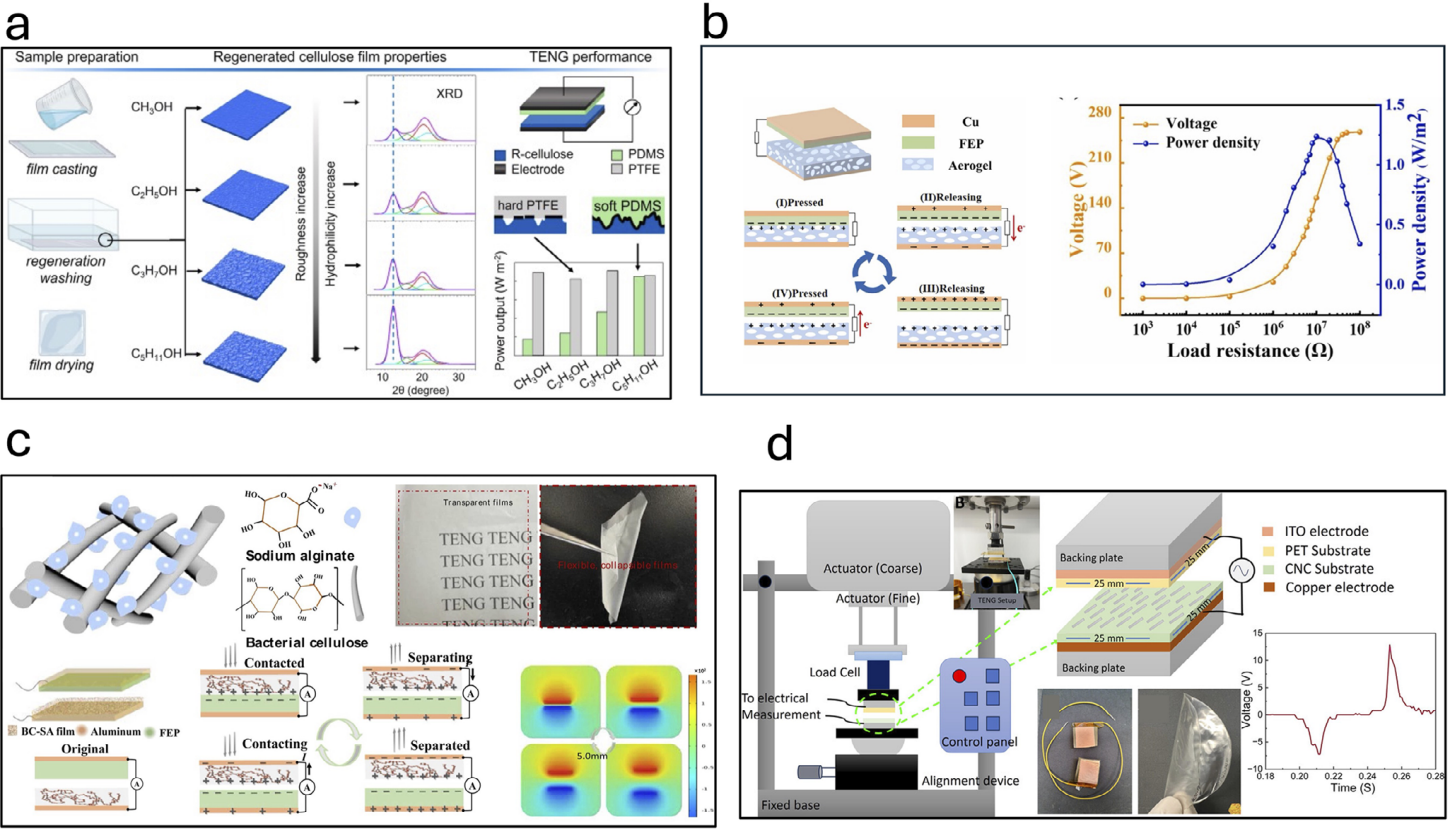
Cellulose materials for TENGs. (a) Regenerated cellulose from different solvents for use as a triboelectric material. Reused from ref. [17] under CC BY 4.0. (b) Bendable substrates of cellulose nanocrystals for TENGs. Reused from ref. [53] under CC BY 4.0. (c) Schematic illustration of the cellulose carbon nanotube aerogel TENG (CCA‐TENG) structure and working mechanism of the contact‐separation mode CCA‐TENG. Reused with permission from ref. [54]. Copyright 2023, Elsevier. (d) TENG based on bacterial cellulose and sodium alginate, with the introduction of BaTiO_3_ nanoparticles. Reused with permission from ref. [55]. Copyright 2024, Elsevier.

### 3.3 Nanocelluloses

Nanocellulose offers a high specific surface area, controllable porosity, and tunable mechanical compliance [56]. Cellulose nanocrystals (CNCs) and cellulose nanofibers (CNFs) produce films with large contact areas and abundant trap states, which are favorable for high surface charge density [57]. Due to the nano‐sized fibers, the thicknesses of the CNC and CNF films are thinner than those of films made of microfibers, which could enhance the performance of C‐TENGs, as the thickness of the dielectric layer can affect the electrostatic induction. Moreover, the chemical structure of cellulose allows the fibers to be hybridized with two‐dimensional sheets such as MXene [12] and graphene [58], which introduces interfacial polarisation and raises the effective dielectric constant, further increasing charge transfer and retention (Figure 3b) [54]. Bacterial cellulose (BC) contributes exceptional purity and wet strength, enabling stable devices in humid conditions when combined with barrier coatings. BC has been utilized in TENGs [21], for example, to fabricate a flexible and transparent TENG for biomechanical energy harvesting [55]. The general preparation of BC films for TENG applications involves a multi‐step process from biomass extraction to film formation, often utilizing scalable methods like casting or filtration.

The initial step for all types is the extraction of nanocellulose from its source material [59, 60]. CNCs are typically isolated through sulfuric acid hydrolysis of microcrystalline cellulose, which removes amorphous regions to yield highly crystalline, rod‐like nanoparticles [61]. CNFs produced via mechanical methods such as high‐pressure homogenization or grinding usually require chemical or enzymatic pre‐treatments (e.g., TEMPO‐mediated oxidation) to facilitate fibrillation and preserve their structure [59, 62, 63]. In contrast, BC is produced by the biosynthesis of specific bacteria, such as *Komagataeibacter xylinus*, in culture medium, followed by extensive purification to achieve high purity [21, 64].

Following extraction and purification, film formation is crucial for TENGs, as both the surface topography and film thickness significantly influence the performance. A common method for producing CNC films is solvent casting or evaporation casting, where the aqueous suspension is poured into a mold and dried, yielding compact and mechanically strong films [65]. Additives, such as triethanolamine (TEOA) and methylimidazole (MI), can be used to improve the mechanical properties in flexible [53]. CNF and BC films are often fabricated by vacuum filtration, which yields flexible, transparent thin films with nanoscale surface roughness, beneficial for TENG performance [66, 67].

A key strategy to further enhance TENG output is to composite nanocellulose with other materials. For example, by compositing nanocellulose with high‐dielectric‐constant particles, such as barium titanate (Figure 3c) and carbon nanotubes (Figure 3d), can increase the dielectric constant and enhance charge transfer and retention, thereby boosting the output of TENGs [54, 58, 68]. These preparation methods enable the development of high‐performance, flexible, and eco‐friendly TENGs for different applications, including self‐powered sensors and wearable electronics.

### 3.4 Modified and Functionalized Cellulose

Surface functionalization can tune both the sign and magnitude of tribo‐polarity in cellulose‐based TENGs by adapting interfacial chemistry, surface potential, and dielectric properties. Oxygen plasma or corona activation is commonly used as a physical method to increase surface energy, introduce hydroxyl/carbonyl functionalities, and etch the surface, thereby moving cellulose toward the tribo‐positive side of the triboelectric series. Such methods can also improve the wettability and contact conformity; however, the same polar groups and increased hygroscopicity can accelerate humidity‐assisted charge dissipation unless followed by hydrophilization or barrier coatings [59, 69].

Covalent derivatization via esterification (e.g., acetylation, succinylation) and salinization provides greater control over polarity. For example, esterification has been shown to reduce hydrogen‐bond density and increase surface hydrophobicity, thereby lowering water uptake. Alkyl‐ or fluorosilane modification creates oriented dipoles that shift the interfacial work function, increasing charge density and improving retention at specific humidity [59, 70, 71]. Amination, such as epoxide/amine coupling, or carboxylation (via TEMPO‐mediated oxidation), increases interfacial hydrogen bonding, thereby enhancing charge transfer. However, one needs to be careful to use the method because ionizable groups may attract ionic screening under humid conditions [59, 69, 70, 71].

Fluorination is another way to tune the triboelectric effect of cellulose, which can be done either by direct introduction of ─CF_x_ groups or by ultrathin fluoropolymer grafts, which raises electron affinity and suppresses surface states that enable back‐transfer, pushing the material toward strong tribo‐negative behavior and higher *V*_oc_ at a given gap distance [72]. First‐principles analyses and systematic experiments on dipole‐engineered polymers show that electron‐withdrawing groups (–CF3) lower interfacial energy levels [72].

In practice, the triboelectric effect of cellulose has been demonstrated to be adaptable through various modifications and functionalizations [41] (Figure 4a). Amination of cellulose can significantly increase the charge density of cellulose from 8 to 18 µC/m^2^ [73], as the ability to lose electrons during contact electrification is enhanced by the amination (Figure 4b). The modification of triethoxy‐1H,1H,2H,2H‐tridecafluoro‐*n*‐octylsilane on cellulose changes the surface polarity and hydrophobicity [18], which retains 70% of the initial output at a high humidity of 70% RH (Figure 4c). The incorporation of polar sulfonic acid groups increases the deep‐trap density at the CNF surface [74], resulting in a nearly 200% increase in surface potential (Figure 4d). The nitrated sisal cellulose exhibits a power density that is over three times that of native cellulose [75], which is attributed to the change in dielectric constant (Figure 4e). Cyanoethyl‐modified cellulose increased the output of the TENG by up to 8 times, and enhanced the charge density at a humidity of 95% to 533 µC/m^2^ [76] (Figure 4f). A new Janus‐type cellulose [77] has been developed recently and used as a triboelectric material that has a low surface wettability, suppressing water‐induced damage to the cellulose network. The asymmetric wettability of the materials exhibits a 917% enhancement in wet strength. Taking advantage of dual‐layer functionalization with MXene and TiO_2_, superhydrophobic cellulose materials [78] have been developed that can maintain the output of TENG at 95.16% at a humidity level of 99%.

**FIGURE 4:**
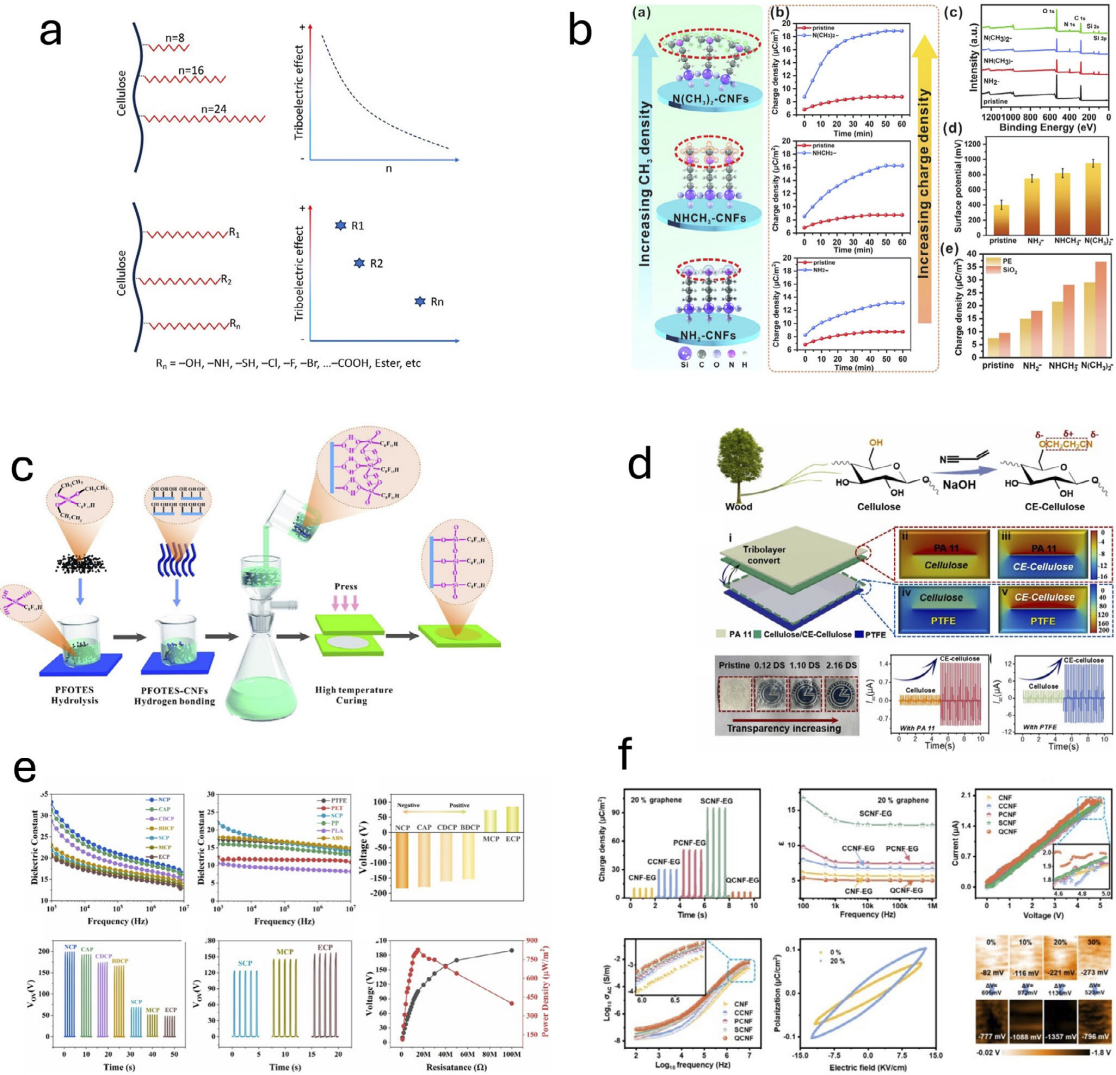
Modification of cellulose for TENGs. (a) Modification to the side chain of cellulose with the same type of molecule but with different lengths of carbon chain. Reused from ref. [41] under CC BY 4.0. (b) Aminated cellulose for TENGs. Reused with permission from ref. [73]. Copyright 2021, Elsevier. (c) Triethoxy‐1H,1H,2H,2H‐tridecafluoro‐*n*‐octylsilane modified cellulose for TENG. Reused with permission from ref. [18]. Copyright 2021, Elsevier. (d) Cyanoethyl cellulose‐based TENG. Reused with permission from ref. [74]. Copyright 2022, Elsevier. (e) Polar groups modified cellulose for TENGs. Reused with permission from ref. [75]. Copyright 2022, Elsevier. (f) Dual interfacial polarized cellulose for TENGs. Reused with permission from ref. [76]. Copyright 2025, Elsevier.

Surface functionalization typically results in changes in surface topography because plasma or wet‐chemical steps can degrade or modify surface molecules. Such changes further alter the active contact area during triboelectrification processes. Either route can enhance the transferred charge per cycle if load and frequency are matched to the viscoelastic response of the modified film.

Ideally, all fluorination‐based functionalization should be confirmed by in situ probes of surface potential and charge decay under controlled humidity/temperature, which can guide the optimization of the final product. Kelvin probe force microscopy (KPFM) [79, 80] may reveal whether gains arise from electron transfer, ion mediation, or polarisation displacement. In short, the most durable improvements pair dipole‐engineering (fluorination, esterification, and amination/salinization, as mentioned above) with moisture management (encapsulation and edge sealing), yielding greater charge density without compromising the material's recyclability and green credentials. Table 1 briefly summarizes the effect of different modifications to the structure of cellulose materials and the functions these modifications bring to C‐TENGs.

**TABLE 1:** The effect of modifications to the structure of cellulose and their functions in C‐TENGs.

Modification	Structural effect	Functions
Amination	More electron‐donating amine Altered interfacial dipoles	More tribo‐positive behavior Enhanced polarization/trapping
Quaternization	Permanent ionic groups	Strong positive polarity Ionic polarization
Esterification/alkyl‐silane	Greater hydrophobicity	Less humidity sensitive Altered polarity and charge retention
Fluorination	Electron‐withdrawing surface groups Reduced wettability	More tribonegative Better moisture resistance
CNT/graphene/MXene fillers	Interfacial polarization Increased effective permittivity	Higher effective dielectric response Higher charge‐storage capability

## 4 Engineering Cellulose Materials for TENGs

To replace engineering polymers in practice, engineering the production of cellulose materials is crucial. A recent study [19] on triboelectric nano‐graphite‐coated paper (NG@P), utilizing commercial materials and an industrial‐scale roll‐to‐roll coating machine [81], has demonstrated the feasibility of commercializing cellulose‐based triboelectric materials (Figure 5). In this report, a thin layer of nanographite (16 µm thick) is coated onto paper at 25 m/min, serving as an electrode layer that replaces commonly used metal electrodes. The resistivity of the nanographite layer has reached 1.037 mΩ m, demonstrating performance comparable to that of a copper electrode in TENGs. According to commercial sources (https://www.alibaba.com/showroom/graphite‐price‐per‐kg.html), the price of graphite ranges from $300 to $1000 per ton. For our NG@P, the weight of nano‐graphite per square meter is 10–15 g, giving a total nano‐graphite weight of 0.0294 kg/m^2^. That said, the cost of graphite per square meter ranges from 0.003 to 0.01$. The price of copper is about $4000–$9000 per ton. Regular copper tape has a thickness of about 0.06–0.1 mm, which gives a price of about $2.14–$8.06 per square meter. For aluminum, the price is about 1000–3500$ per ton, which gives a price per square meter of 0.08–0.28$ for a tape with a thickness of 0.03 mm. In summary, the cost of graphite per square meter in our study is lower than 1/8 of aluminum tape and 1/220 of copper tape. The price of Kraft paper per square meter is about 0.002$, which puts the total material cost in the range of 0.005–0.012$. Therefore, it can be confirmed that NG@P is a cheaper alternative to metal electrodes.

**FIGURE 5:**
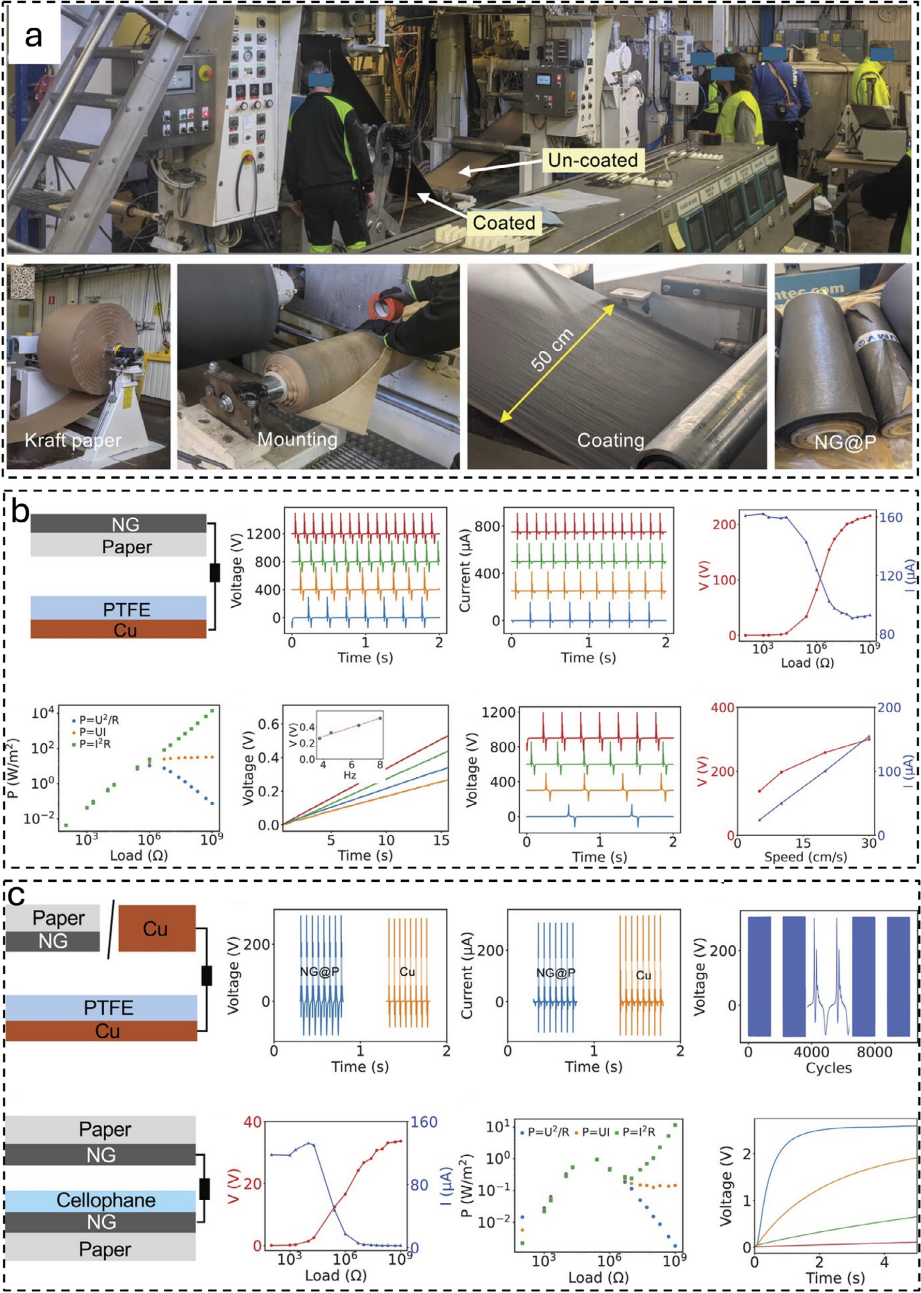
Engineering triboelectric paper for C‐TENGs. (a) Photographs of the coating of nanographite on Kraft paper using an industrial coating machine. (b) Characterizations of NG@P based TENGs. (c) Comparison of the nanographite electrode with the copper electrode, and the experiment uses NG@P as both the triboelectric layer and the electrode. Figures reused from ref. [19] under CC BY 4.0.

Regenerated cellulose, which has been proven to have a strong positive charge affinity [15], may be engineered as triboelectric materials. This is possible because the regeneration process can be easily scaled up. Commercially, regenerated cellulose films are available, but they cost much more than Kraft paper due to the small market. There is a need for more C‐TENGs based on regenerated cellulose for sale to boost production and, subsequently, reduce prices. Opportunities also arise from reduced plastic use, which may stimulate the production of regenerated cellulose that can benefit C‐TENGs.

For other cellulose materials, such as CNF and BC, their engineering for triboelectric applications is not only based on their production but also on their composition with other materials. The reason is that CNF and BC, as triboelectric layers, do not provide C‐TENGs with high performance. They need to be composited with other materials, as shown in Table 1, to increase the electric output of the C‐TENGs.

## 5 Applications of C‐TENGs

C‐TENGs have been used in many applications ranging from energy harvesting, wearable electronics, self‐powered tactile sensors, HMI, gas sensors, etc. Because the applications are different, there are different needs for cellulose materials to fulfil the requirements. Table 2 summarizes the key requirements for cellulose materials for different applications, as well as their advantages and limitations.

**TABLE 2:** Material requirements for cellulose materials for different applications, and their advantage and limitations.

Application	Key requirements	Cellulose‐specific advantages	Major limitations
Energy harvesting	Charge density Dielectric strength Fatigue resistance	Low mass Flexibility Renewable Scalable sheet/film processing	Humidity sensitivity Protocol‐dependent output
Wearable electronics	Compliance Breathability Low‐force sensitivity Sweat stability	Porosity Conformability Textile/paper compatibility	Moisture uptake Long‐term drift
Tactile/HMI	Sensitivity Spatial resolution Hysteresis Array uniformity	Foldability Printability Low‐cost	Batch variability Wear
Air filtration	Filtration efficiency Pressure drop Charge retention, RH stability	Hierarchical porous networks Compatibility with functional additives	Charge decay under high humidity
Gas/food sensing	Selectivity Detection limit Response/recovery speed RH cross‐sensitivity	High surface area Hydroxyl chemistry Paper‐compatible packaging	Humidity interference Dominant receptor may be a non‐cellulosic additive
Healthcare	Biocompatibility Sterilization Mechanical compliance	Softness Breathability Conformability	Fluid ingress and long‐term stability
Smart transportation	Mechanical robustness Positional accuracy Environmental tolerance	Compatibility Low‐cost arrays Scalable integration	Wear Moisture drift

### 5.1 Energy Harvesting

Table 3 summarizes the material pairs and electrical output of C‐TENGs, as well as their stabilities tested in the reported works. The energy sources to be harvested by C‐TENGs range from body motions, wind or airflow, raindrops, to mechanical vibrations. Depending on the types of cellulose material and the pairing triboelectric material, the output of C‐TENGs falls in a wide range. The open‐circuit voltage of C‐TENGs is in a range of several volts to more than 1000 volts, and the short‐circuit current is in a range of several hundred nanoamperes to several milliamperes. The highest power density is 14 kW/m^2^, which is due to electrostatic discharge on the paper surface [19]. For CNC, CNF and BC, the energy output of the C‐TENGs made of their composites is higher than that made of the materials themselves, because the composition increases the triboelectric properties that have been described in the section above. Paper‐based C‐TENGs exhibit better performance than CNC‐, CNF‐, and BC based C‐TENGs, which is due to surface modification of the paper that usually changes the surface hydrophobicity.

**TABLE 3:** The performance of C‐TENGs. Detailed abbreviations of the materials can be found in the original references.

Cellulose material	Counter tribo‐layer	*V*_OC_	*I*_SC_	Power/power density	Stability (cycles)	Refs.
Treated wood	PTFE	81 V	1.8 µA	51 µW (0.057 W/m^2^)	20 000	[82]
BNC	Copper (BNC‐bio TENG) Polyimide (BNE‐PI TENG)	13 V 7 V	11 µA NA	0.0048 W/m^2^ NA	NA NA	[21]
Tissue paper	Glass Printer paper Cotton fabric Wood PET PVC	17 V 40 V 25 V 20 V 100 V 100 V	NA	NA	NA	[83]
PFOTES‐CNF	Polyamide	28.5 V	9.3 µA	0.0135 W/cm^2^	10 000	[18]
CNF‐phosphorene	PET	5.2	1.8 µA	1.1	500	[84]
Methyl‐CNF	Nitro‐CNF	8	9 µA	—	500 000	[85]
CNF aerogel	Porous polyimide	60.6	7.7 µA	2.33 W/m^2^	10 000	[86]
CNF‐PEI aerogel	PVDF	106.2	9.2 µA	13.3 W/m^2^	10 000	[87]
CNF‐PEI‐Ag	FEP	100 (one pair) 286 (Three pairs)	1.1 µA 4 µA	NA 0.43 W/m^2^	10 000	[88]
Alc‐S_5_‐CNF	PVDF	7.9 V	9.3 µA	0.101 W/m^2^	7000	[89]
Paper	PTFE	400 V	0.17 mA	53 W/m^2^	—	[90]
Rice paper	PVC	244 V	6 µA	0.376 W/cm^2^	10 000	[91]
Fluorinated cellulose	Cellulose	94 V	8.5 µA	0.15 W/m^2^	15 000	[92]
CNF	FEP	30 V	90 µA	0.56 mW	500 000	[27]
Ppy/cellulose	Nitrocellulose	60 V	8.8 µA	0.83 W/m^2^	10 000	[93]
Crepe cellulose paper	Nitrocellulose	196.8 V	31.5 µA	16.1 W/m^2^	10 000	[94]
Cellulose II aerogel	PTFE	65 V	1.86 µA	0.127 W/m^2^	15 000	[95]
Gum wrapper paper	PVDF	1000 V	30 µA	NA	21 000	[96]
TCA‐MBT‐PVDF	Polyamide	1040 V	1.165 mA	4.2 W/m^2^	100 000	[97]
CNF	FEP	155 V	17.5 µA	0.22 W/m^2^	10 000	[98]
Lignocellulosic CNF	Cu Al	300 V 360 V	25 µA 28 µA	0.52 W/m^2^ NA	72 000	[22]
BC/HEC aerogel	PVDF	21 V	0.39 µA	0.0079 W/m^2^	6000	[99]
ε‐PL‐TOCN	VHB	4.66 V	NA	NA	1800	[100]
MWCNT/CNF/PVA hydrogel	Cellulose paper	160 V	1.74 µA	NA	2000	[101]
Chitosan/di‐aldehyde NC	FEP	314 V	30 µA	0.2 W/m^2^	40 000	[102]
Modified cellulose	Kapton	335 V	9.74 µA	3.8 W/m^2^	20 000	[103]
Waste paper	FEP	774 V	3.92 mA	43.5 W/m^2^	2000	[28]
BC‐SA	FEP	530.1 V	0.62 µA/cm^2^	1.81 mW	600	[55]
BC/CNT/PPy	PDMS	170 V	0.8 µA	0.352 mW	1000	[104]
BC‐CNT‐PPy	BC	29 V	0.6 µA	0.003 mW	10 000	[23]
Nano graphite/paper	PTFE	318 V	164 µA	14 kW/m^2^	10 000	[19]
Paper	Teflon	85 V	3.75 µA	0.398 W/m^2^	NA	[105]
CNC oCNC	Nitrile PTFE	13 V 12.3 V	72 nA 27–700 nA	0.0011 W/m^2^ 0.16 W/m^2^	NA NA	[106]
Sisal cellulose	PTFE	118 V	6.13 µA	2.56 kW/m^2^	2000	[75]
Paper	Teflon	326 V	45 µA	5.42 W/m^2^	10 000	[107]
Ethylcellulose	Stainless steel	245 V	50 µA	NA	NA	[108]
Ethylcellulose	PDMS	45 V	NA	0.0012 W/m^2^	1000	[109]
Regenerated cellulose	Cellophane	300 V	2.6 mA	300 W/m^2^	30 000	[15]
Cellulose acetate	PTFE	7.3 V	9.1 µA	NA	300	[110]
Cellulose acetate/PEI	Silicone rubber	478 V	6.3 µA/cm^2^	0.0022 W/m^2^	10 800	[111]
BC/Ag Np/BTO	PTFE	400 V	23 µA	1.8 W/m^2^	10 000	[112]

In all the C‐TENGs, cellulose materials in different forms have different roles but not only as an active triboelectric layer. However, the roles are not clearly described in all publications. Here, we summarize the roles in Table 4 and briefly describe the functions of these roles.

**TABLE 4:** Roles of cellulose materials in C‐TENGs.

Role of cellulose material	Function
Active triboelectric layer	Cellulose directly participates in contact electrification and contributes materially to net charge transfer.
Dielectric/charge‐storage matrix	Cellulose mainly controls dielectric response, trapping, field distribution, or charge retention.
Scaffold/host	Cellulose supplies porosity or mechanical integrity for a functional additive that performs the dominant sensing/tribological function.
Substrate/support	Cellulose mainly carries or mechanically supports the functional triboelectric layer or electrode.
Filtration or packaging medium	Cellulose provides porous structure, breathability, or packaging integration while the functional response may originate elsewhere.

### 5.2 Wearables and Body‐Motion Energy Harvesting

C‐TENGs have been widely explored as wearable‐friendly devices where the cellulose materials in the C‐TENGs can be engineered into breathable membranes or films that conform to the skin. For example, a microbial cellulose‐based TENG has been developed for long‐term on‐skin contact, thanks to its high breathability and antibacterial properties, which can harvest energy from joint motion to power small electronics [113]. Another example of taking advantage of the breathability of cellulose materials is a TENG‐based face mask made from air‐permeable cellulosic triboelectric materials. Such a face mask can not only harvest energy from respiratory airflow and facial motions but also provide high‐efficiency PM filtration, which highlights the dual role of cellulose as both a mechanical transducer and a functional filter medium in TENGs [114]. By modifying cellulose materials, researchers have made a biodegradable, flame‐retardant cellulose‐based TENG that can be integrated with firefighting clothing. Such a TENG can produce tens of volts, which is enough to drive LEDs and charge capacitors for emergency signals [115].

Researchers have also explored the application of paper as a triboelectric layer, in addition to creating layers from cellulose, due to the large number of available materials. The flexibility of paper enables the design of folding origami patterns for TENGs [116], such as slinky‐ and doodlebug‐like patterns, which can be used to harvest energy from stretching, lifting, and twisting motions of the arm and hand to drive small electronics. Similar to the origami patterns, interlocking kirigami‐patterned TENGs have been developed with excellent stretchability using ordinary office paper. Such TENGs can power a liquid crystal display (LCD) with the energy harvested from elbow and knee motions [117]. Besides the origami and kirigami that allow flexibility through patterns, gel‐based cellulose TENGs can also be flexible and stretchable [118], making them suitable for wrapping around joints for comfortable energy harvesting during large deformations.

Building on the advances in C‐TENGs for wearable and bioelectronic applications under challenging environmental and mechanical conditions [119], hybrid nanogenerators (HNGs) have been developed that combine TENGs and piezoelectric nanogenerators (PENGs). Such an HNG can generate power underwater and at both low and high temperatures, owing to the specific properties of polydopamine@BaTiO_3_/cellulose acetate nanofiber films [68].

### 5.3 Self‐Powered Sensors

#### 5.3.1 Tactile and Pressure Interfaces

The flexibility, lightweight, and foldability of cellulose and paper substrates make them excellent candidate materials for tactile sensors and pressure‐sensing interfaces. Researchers have developed C‐TENG‐based keyboards, pianos, and other devices, opening a new window for interactive paper electronics. In most studies, the C‐TENG‐based tactile sensors are developed for sensing finger or hand touching. Normally, the sensors are attached to a substrate close to another functional unit. Cases of sensors directly attached to fingers are also reported as a wearable sensing system [120].

The C‐TENGs can act as a single signal source [121] or be assembled into an array [122] to provide information on a 2D surface. In the array, each TENG unit generates a distinct voltage signal upon touch or pressure, allowing researchers to map the pressure distribution without an external power supply. Meanwhile, one can correlate the signal intensity, position, and timing with touch or pressing. Such features enable the array to be used as a keyboard or keypad. Experimental results have shown that the arrays’ stability and repeatability are excellent, as evidenced by comparing signals before and after thousands of pressing cycles [94].

From a material standpoint, both cellulose materials [123] and their composites [124] have been used to construct tactile sensors. The operation mode of C‐TENGs for tactile sensing is generally the contact‐separation mode. The performance of the tactile sensors varies across reports, as both linear and nonlinear responses of electrical signals to force or pressure are observed. Table 5 summarizes the materials and the performance of C‐TENG‐based tactile sensors.

**TABLE 5:** C‐TENG‐based tactile sensors.

Cellulose material	Counter material	Operation	Linear range	Sensitivity	Refs.
A‐GC hydrogel	PTFE	Attached to finger			[120]
ES‐CA	BN‐PVP	Adding weight	0.5–2 N 2–10 N	3.98 V/N 1.84 V/N	[121]
CCi/Mxene	FEP	Boxing glove			[125]
BCIG	PDMS	Finger touch			[126]
Nitro‐CNF	Cu	Motor	0.5–3 V cm^2^	8.276 V cm^2^/N	[124]
CMC‐PDMS	Al/Cu	Body motions			[127]
Cellulose/NaCl hydrogels	Dielectric film		∼150 kPa	0.0068/KPa	[128]
Cellulose acetate n‐nofibers (CANF)	PDMS	Force impactor	0.05–1 N 1–10 N	∼3.67 V/N 1.073 V/N	[123]
Ag@CF	PTFE	Coins	3.2–9.1 g (estimated from figure)		[129]
Paper	PTFE	Linear motor	Non‐linear		[116]
Nitrocellulose	Microstructure cellulose film	Universal material testing machine	Non‐linear		[130]
PHMS@PCP	PA	Hand			[131]
rGO CNT paper	Cu	Universal material testing machine	0–5 kPa	18.6782 /kPa	[132]
CPHP	Cu	Linear motor	0–16 kPa	3.95 mV/Pa	[122]

#### 5.3.2 Motion, Posture and Gait Sensing

C‐TENGs have been widely studied for sensing body motion, posture, and gait due to their mechanical flexibility, breathability, and compatibility with wearable systems [133, 134, 135]. Compared with engineering polymer‐based TENGs, cellulose offers improved air permeability and moisture management, which are of great importance for long‐term wearability and user comfort [136, 137]. Table 6 summarizes some of the applications of C‐TENGs for motion sensing.

**TABLE 6:** C‐TENGs based motion, posture and gait sensing.

Cellulose material	Counter material	Motion sensing	Demon application	Refs.
Crepe cellulose paper	Nitrocellulose	Finger tapping	Paper piano	[94]
PEO/CCP	PDMS	Respiration, pulse		[138]
Polydopamine/CNF	FEP	Clapping, clicking, skipping, walk		[139]
PVDF/CFP	PI	Jump, run, walk, tap, slide, bend	Motion identification	[140]
GO/CMC	FEP	Tapping, bending		[58]
Milk paper	PTFE	Tapping, walking, running, jumping		[141]
Kraft paper		Walking, arm and leg motions, respiration	Smart sheet	[19]

Early studies on paper‐based TENGs have demonstrated the feasibility of sensing simple touch and finger motions and have shown their application in human–machine interactions (HMI). An example is the development of all cellulose‐based TENGs for sensing finger tapping [94], where crepe cellulose paper and nitrocellulose membrane are used as triboelectric pairs. To improve motion‐sensing resolution and durability, cellulose composite materials have been developed, in which polymers such as polyethylene oxide (PEO) [138], polydopamine, and PVDF have been used to composite with cellulose (Figure 6a). The compositing of cellulose with PEO significantly increases the sensitivity of the TENG sensor, enabling it to measure physiological signals such as eye muscle motion, respiration, wrist pulse, and heartbeat. The composition of cellulose nanofibrils with polydopamine has been found to increase surface polarity and surface charge density, as well as the effective contact area, by creating polydopamine particles [139]. These enhancements enhance the performance of TENGs made from materials that can sensitively detect human motions, such as skipping, walking, and clapping. By immersing cellulose filter paper (CFP) in a PVDF solution, a PVDF/CFP composite film has been formed, exhibiting a stronger triboelectric effect than the CFP itself, which allows the recognition of multiple human actions with the assistance of deep learning [140]. A KPFM study has shown that GO binding to carboxymethylcellulose can increase the surface potential/charge, as GO alters the electronic states of carboxymethylcellulose. Such an increase in surface charge enables the measurement of bending at different joints, such as the finger, elbow, and knee [58].

**FIGURE 6:**
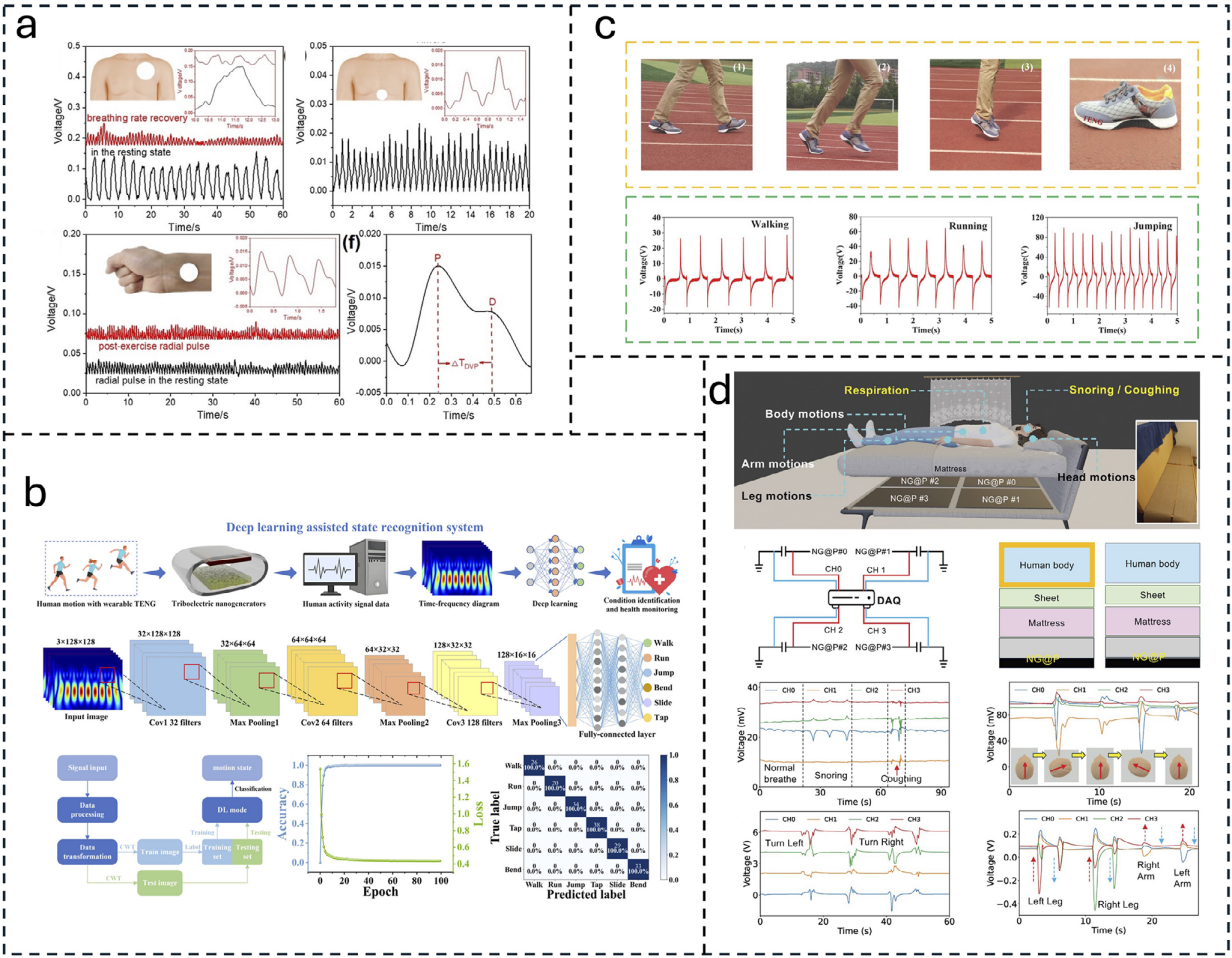
C‐TENG‐based motion sensing. (a) Physiological information obtained from the PEO/CCP composite constituted the C‐TENG sensor. Reused from ref. [138] with permission. Copyright 2023, Elsevier. (b) Deep learning‐assisted TENG for human motion recognition based on a C‐TENG. Reused from ref. [140] with permission. Copyright 2025, ACS. (c) Human motion states such as (1) walking, (2) running, and (3) jumping; (4) shoe with installed MP‐TENG. Reused from ref. [141] with permission. Copyright 2019, Elsevier. (d) Sensing body motions with a smart sheet made of nanographite‐coated paper. Reused from ref. [19] under CC BY 4.0.

The roles of cellulose materials, such as paper, can extend beyond serving as triboelectric materials to also acting as supporting materials. A milk layer can be coated on paper origami to sense various motions (Figure 6c), such as walking, running, jumping, and limb movement, while simultaneously harvesting energy from these motions [141]. From the output, one can obtain information such as the pressure and frequency of the body movements, which could be useful for activity tracking and motion monitoring.

Wireless motion sensing and tracking based on cellulose triboelectric materials have also gained rising interest in the last several years. The working mechanism is the capacitance change between the human body and the sensor. The triboelectric potential generated on the human body changes in response to body motions, such as walking, which alters the capacitance and results in a measurable voltage change [19]. By increasing the number of sensors in a sensing array [57], it is possible to detect human walking and provide two‐dimensional data, including direction, speed, and position. If the array is placed under the mattress of a bed [19], it is reported to sense both motion in bed and the physiological activities such as breathing and snoring (Figure 6d).

#### 5.3.3 Human–Machine Interfaces

C‐TENGs can enable HMI by converting body motion into electrical signals that serve as control inputs. Compared to other HMI systems, these C‐TENGs function as energy harvesters, sensors, and signal sources, enabling self‐powered interfaces [142, 143]. Results from studies have shown that cellulose materials can be used as either a triboelectric layer or a supporting substrate to provide a reliable signal, even under low pressure or subtle user interactions, which enables the construction of HMIs.

The most studied TENG–HMI mode is touch‐ and press‐based interaction, such as those found in paper keyboards and pianos [136]. In these devices, arrays of C‐TENG are designed so that each button corresponds to a distinct electrical channel. The C‐TENG can operate in either the contact‐separation or single‐electrode mode, and the voltage signals are typically used to trigger specific commands, such as musical notes [94] and keystrokes (Figure 7a) [144]. The arrays are normally mounted on a substrate [145], but they could also be attached directly to fingers for HMI (Figure 7b) [146]. Because the C‐TENGs in the arrays are connected to distinct channels, there is no need for machine learning to process the electrical signals, thereby simplifying communication between body motions and machine actions.

**FIGURE 7:**
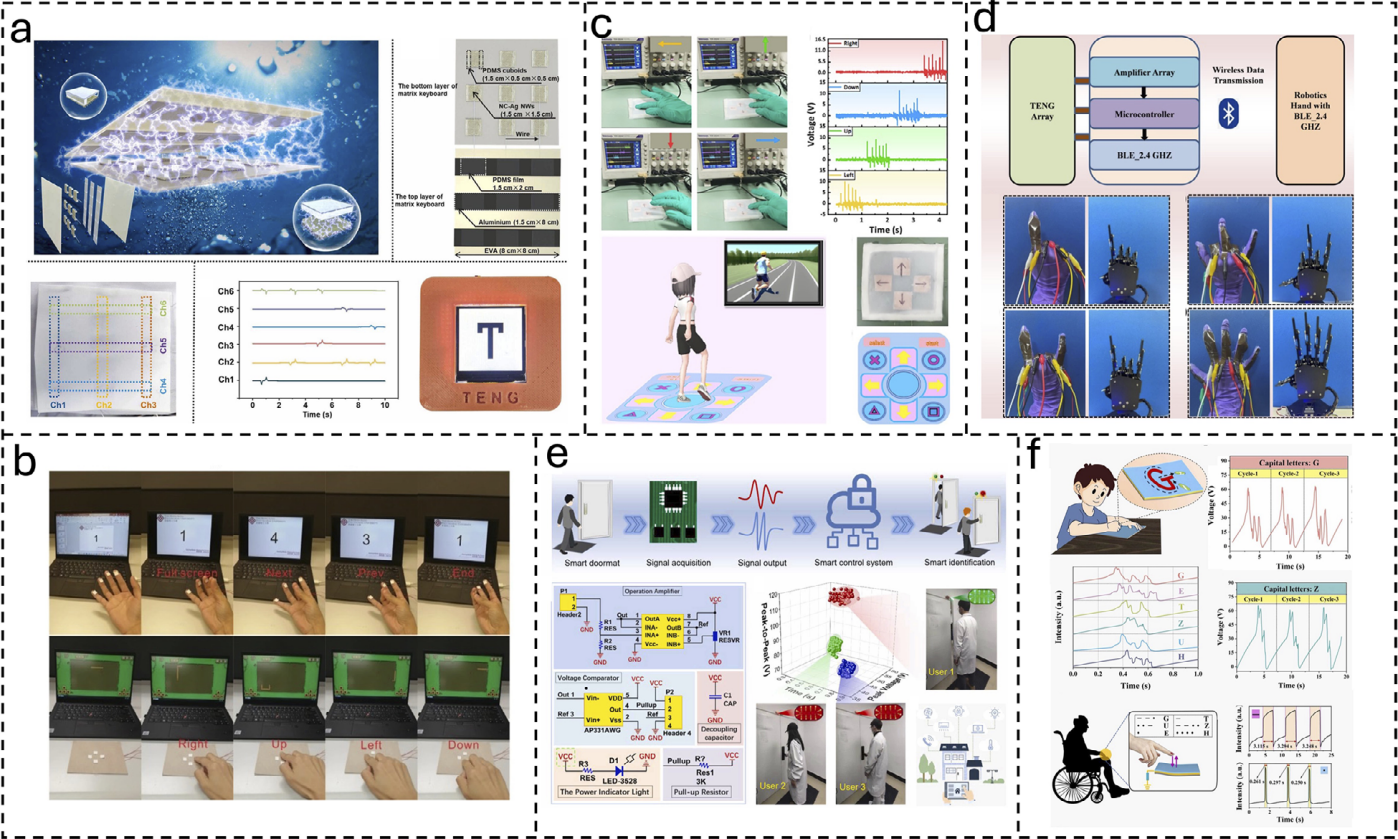
C‐TENG‐based human–machine interfaces. (a) A 3 × 3 matrix keyboard made of nitrocellulose‐based TENG. Reused from ref. [144] with permission. Copyright 2023, Elsevier. (b) Recycled cellulose‐based TENG sensors attached directly to fingers for a human–machine interface. Reused from ref. [146] with permission. Copyright 2022, Elsevier. (c) Conductive fabric‐based HMI for game control. Reused from ref. [145] with permission. Copyright 2023, ACS. (d) Grass/Gum TENG‐based HMI for robotic control systems. Reused from ref. [147] with permission. Copyright 2025, Elsevier. (e) Papermaking‐inspired TENG for HMI using signal waveforms for analysis. Reused from ref. [148] with permission. Copyright 2024, Elsevier. (f) Text input without the assistance of machine learning‐based C‐TENG sensors. Reused from ref. [149] with permission. Copyright 2024, Elsevier.

Besides discrete touch inputs, gesture‐ and motion‐based HMIs have also been experimentally demonstrated. To capture the motions, C‐TENGs are attached to fingers, wrists, or shoes to generate signal patterns that correlate with motion types, directions, frequencies, and intensities. The signals are interpreted to recognize gestures, gait, etc., enabling HMIs such as smart carpets, game controllers (Figure 7c) [145], robotic control systems (Figure 7d) [147], and multimedia controllers. Machine learning or deep learning algorithms, such as convolutional neural networks (CNN) [140], are commonly used to enhance the efficiency and accuracy of recognition, because the waveform of the signals is more complex than simple threshold detection for keystrokes. However, the waveform of the signals contains much more information, allowing for more interactions at the HMIs [148].

Compared to gesture‐based HMIs, handwriting is a more natural method of text input on C‐TENGs. Here, one can convert recorded electrical signals into letters using either intensity or waveform information. Depending on the intensity, a signal can be interpreted as a dash or a dot of Morse code, and a combination of several signals can be interpreted into a letter. If the HMI is based on the signal waveforms (Figure 7e) [148], parameters such as the number of peaks, peak width, signal period, etc., should be considered [150, 151]. In practice, depending on the aims, researchers have used text input with [140, 152, 153] or without [131, 149, 154, 155, 156] (Figure 7f) the assistance of machine learning.

Typically, TENG‐based text input strategies are accomplished by hand or finger motion, which involves time‐varying triboelectrification in a specific position. Recently, a paper‐supported TENG‐based mask has been invented for text input via breathing [157], in which long and short breaths are recorded as the dash and dot of Morse code, respectively, and translated into letters. Such a strategy can help people with specific disabilities improve their quality of life.

#### 5.3.4 Medical and Healthcare Applications

Medical and healthcare applications of C‐TENGs have garnered increasing interest in recent years, as cellulose materials are environmentally friendly and renewable. These types of applications include: targeted cancer treatment, respiratory monitoring, implantable medical devices, etc.

The electric field generated by a TENG made of a polylactic acid (PLA) film and a reed film has been found to stimulate in vivo drug delivery, where the release of doxorubicin can be accelerated by the electric field (Figure 8a). The application of the TENG enhanced the cytotoxicity against cancer cells [158]. Such an application has great potential to be extended to handle other drug release‐related issues, such as addressing drug resistance by controlling the release dynamics with a C‐TENG.

**FIGURE 8:**
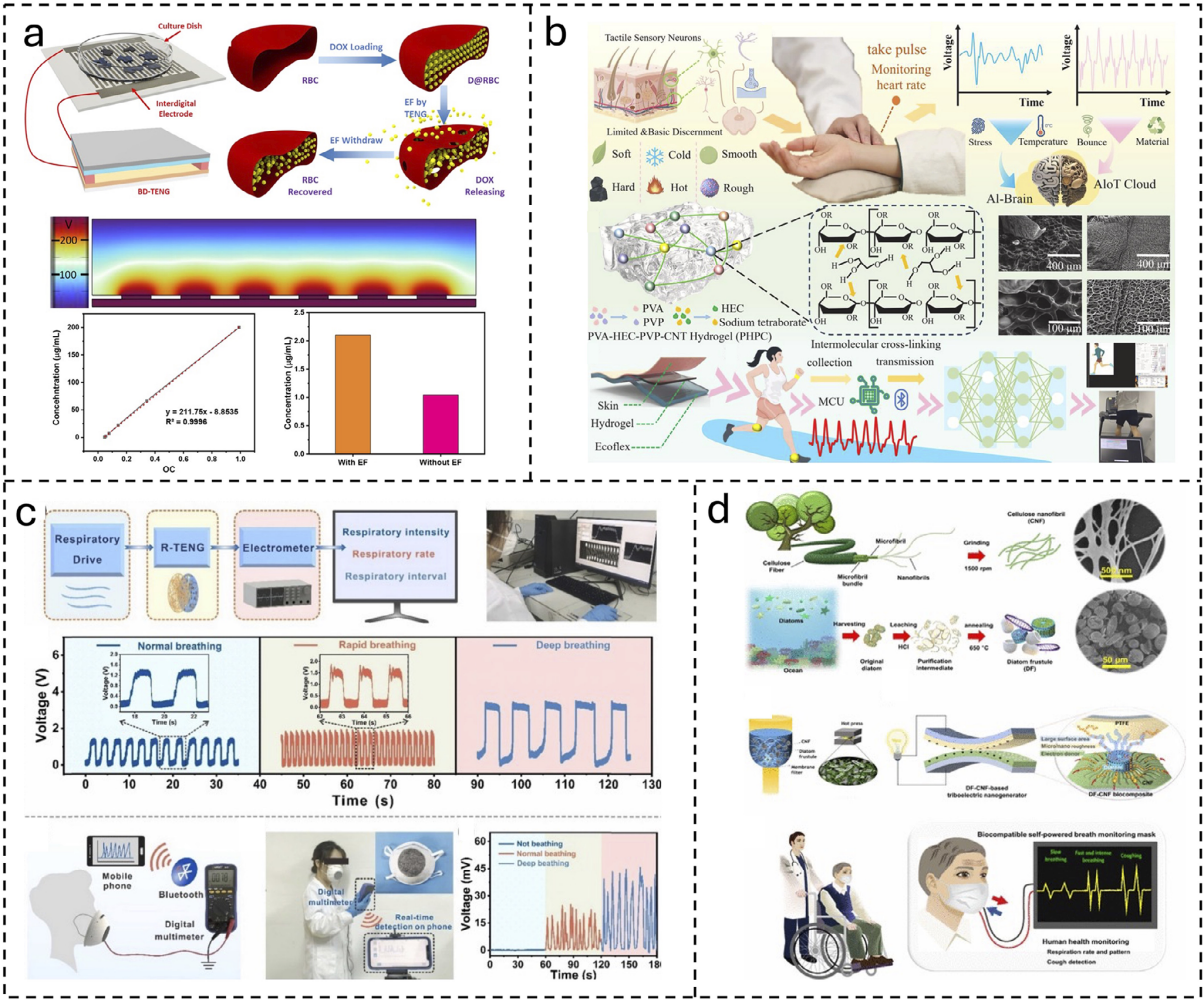
C‐TENGs for medical and healthcare applications. (a) TENG made of a polylactic acid (PLA) film and a reed film for enhancing cytotoxicity. Reused from ref. [158] under CC BY 4.0. (b) Hyperstretchable cellulose‐based hydrogel TENG pulse sensing. Reused from ref. [159] with permission. Copyright 2026, Elsevier. (c) Cellulose aerogel/Ni‐HITP based respiration‐driven TENG for breathing monitoring. Reused from ref. [114] with permission. Copyright 2022, Elsevier. (d) Cellulose nanofibril film‐based TENG for respiratory sensing. Reused from ref. [160] with permission. Copyright 2021, ACS.

The monitoring of cardiovascular health has been studied by using engineering polymer‐based TENGs. Recently, a hyperstretchable cellulose‐based hydrogel TENG has been developed for sensing pulse at the carotid artery [159], where the intensity of the triboelectric potentials is correlated to different states of exercise (Figure 8b). Similarly, a PEO/cellulose composite paper‐based TENG has been shown to sense heartbeats and write pulses [138].

Respiratory sensing and monitoring are other important applications of C‐TENGs that can provide physiological information about people. The most straightforward form of respiratory monitoring is achieved with mask‐shaped C‐TENGs, in which triboelectric signals are triggered by airflow through the nose. An example is a cellulose aerogel/Ni‐HITP based respiration‐driven TENG (Figure 8c), which can perform real‐time breathing monitoring and has the capability to distinguish breathing patterns by frequency and intensity [114]. Another example is a cellulose nanofibril film composed of a diatom frustule‐based mask [160], in which a FEP thin film vibrates between two films to generate triboelectric signals (Figure 8d). The vibration of the FEP film is triggered by breathing, and the signals can be enhanced by applying multiple TENGs. Beyond respiratory sensing, mask‐shaped C‐TENGs can also perform air filtration while sensing breathing, as the triboelectric potential on the materials’ surfaces can attract small particles.

Besides the mask‐shaped C‐TENG, other C‐TENG types have been developed for respiratory sensing, each with a different working mechanism. Recently, a sheet‐like paper TENG array has been developed to detect human respiration while people sleep [19]. Despite its complex working mechanisms, the array can detect normal breathing, snoring, and coughing. Moreover, the array is placed under a mattress that does not come into direct contact with the skin or clothing, resulting in high comfort and acceptability. A C‐TENG can also be attached to the abdomen to detect a person's respiration, as the abdomen fluctuates to varying degrees during breathing, and the patterns differ between individuals [161].

#### 5.3.5 Air Filtration and Environmental Monitoring

Utilising the triboelectric potential to attract small particles for air filtration has attracted interest ever since the invention of TENGs. With the development of C‐TENGs, their application for air filtration has also been widely investigated. The air filtration effects of C‐TENGs are usually evaluated by the removal efficiencies for PM particles. Table 7 summarizes the results reported in the articles. For air filtration, the C‐TENGs are mostly made as masks to be worn by users to filter away PM particles while breathing. In these cases, the particle size to be filtered is down to 0.3 µm (PM 0.3), which cleans the air breathed. The overall removal efficiency increases with particle size, as indicated by several studies [114, 162, 163, 164]. Besides masks, radial piston [165] and filter films [166] have also been fabricated that could be embedded in larger systems for air purification in buildings.

**TABLE 7:** Air filtration effects of C‐TENGs. The percentages indicate the removal efficiencies.

Cellulose material	Counter material	TENG shape	PM 0.3	PM 0.5	PM 1.0	PM 2.5	PM10	Refs.
GO/CNF	CBA	Mask	98.97%	99.65%	99.93%			[162]
CD/TEOS/Ti_3_C_2_T_x_/MoS_2_ (CTTM)	(PVDF‐HFP)/BaTiO_3_	Mask	98.72%					[167]
CMFs/CNFs	FEP	Mask				98.83%		[24]
CNF	FEP	Radial piston				83.78%	86.82%	[165]
Cellulose aerogel/MOF	PVDF	Mask	95%	97.3%	98.4%			[114]
CD/TEOS/Ti_3_C_2_T_x_/ZIF‐8 (CTMZ)	(PVDF‐HFP)/BaTiO_3_	Mask	99.21%	99.71%	99.98%			[163]
Ag‐Ligno cellulose	FEP	Filter	98.30%	98.35%	99.70%			[164]
Ag/PDA/CNF	PVDF	Mask	94.23%					[168]
Cyanoethyl cellulose/PVDF	CMC/Zein	Filter				99.96%		[166]

From the reported results, one can observe that cellulose composites with other materials exhibit higher removal efficiency [162, 163] than cellulose alone [24, 165]. Of course, the difference in efficiency may be due to differences in device construction. However, the formation of dipoles by the composite with other materials may be another reason for the enhancement of the removal efficiency.

#### 5.3.6 Gas Sensing

Triboelectrification occurs between the surface molecules of two materials, meaning that any changes to the surface molecules will result in changes in the charge transfer. Such a phenomenon underlies gas sensing based on C‐TENGs, as the binding of gas molecules to surface molecules alters their states, leading to changes in triboelectric signals. Among all the studied gases, ammonia is most extensively studied due to its polarity and the lone pair of electrons on the nitrogen atom, which enables it to interact with other molecules. The lone electron pair allows ammonia to form strong hydrogen bonds with other molecules. Another reason is that ammonia is an important indicator of food quality. Table 8 lists some of the C‐TENGs for gas sensing applications.

**TABLE 8:** C‐TENG‐based gas sensors.

Cellulose material	Counter material	Target gas	Detection limit	Linear range	Sensitivity	Refs.
CD/TEOS/Ti_3_C_2_T_x_/ZIF‐8 (CTMZ)	(PVDF‐HFP)/BaTiO_3_	Ammonia	1 ppm	1—150 ppm	0.00518 V/ppm	[163]
MXene/TiO2/C‐NFs	CA‐NFs	Ammonia		1–100 ppm (logarithm)		[169]
MXene/CeO_2_/cellulose	Silicone	Ammonia		0.1–10 ppm		[170]
PFOTES‐CNF	PA	Ammonia	10 ppm	10–120 ppm	2.1 (*V*_air_/*V*_gas_)	[171]
CPC aerogel	FEP	Ammonia		20–150 ppm	0.00458 V/ppm	[172]
CNF cryogel	PVA‐CNF‐PEO	Ammonia		20–100 ppm	0.16148 V/ppm	[173]
Balsa scaffold/PANI/SnO_2_	FEP	Ammonia	5 ppm	5–200 ppm	0.0038 V/ppm	[174]
ZnO/cotton	PET	Acetone	45 ppm	∼850 ppm (read from figure)	0.15374 V/ppm	[175]
Cellulose acetate	PVDF/TiO_2_	Acetone			0.0035 V/ppm	[176]
Leek out skin	Leek inner skin	Acetone	3.92 ppm	3.92–39.2 ppm 39.2–160 ppm		[177]

An example is the interaction of ammonia with MXene that changes the triboelectric effect of MXene/TiO_2_/C‐NF, where the response is found to be linearly related to the logarithm of the ammonia concentration in the range of 1–100 ppm [169]. Similarly, MXene/CeO_2_/cellulose nanofiber composites (MC/C‐NFs) based ammonia sensors can sense lower concentrations down to 0.1 ppm [170] (Figure 9a). Besides MXene, titanium carbide (Ti_3_C_2_T_x_) is another material that ammonia can bind to. Researchers have suggested that ammonia can bind to the ─O and ─OH groups on the Ti_3_C_2_T_x_ surface, which changes the charge transfer of the composite made of Ti_3_C_2_T_x_ and CNF [163, 171] (Figure 9b). Carbon nanotube (CNT) composited cellulose aerogel can also be used for ammonia sensing (Figure 9c), where the CNT offers binding sites for ammonia and leads to a linear detection range of 20–150 ppm [172]. Using MIL‐125 (Ti_8_O_8_(OH)_4_[O_2_C─C_6_H_4_─CO_2_]_6_) hybrid CNF cryogel (Figure 9d), ammonia can be sensed with the same linear response range of 20–100 ppm [173]. Besides physical binding, the reversible chemisorption of ammonia to PANI can also be utilized for sensing (Figure 9e), where ammonia is ionized while binding to PANI [174]. A detection limit of 5 ppm and a linear range of 5–200 ppm have been observed using this strategy.

**FIGURE 9:**
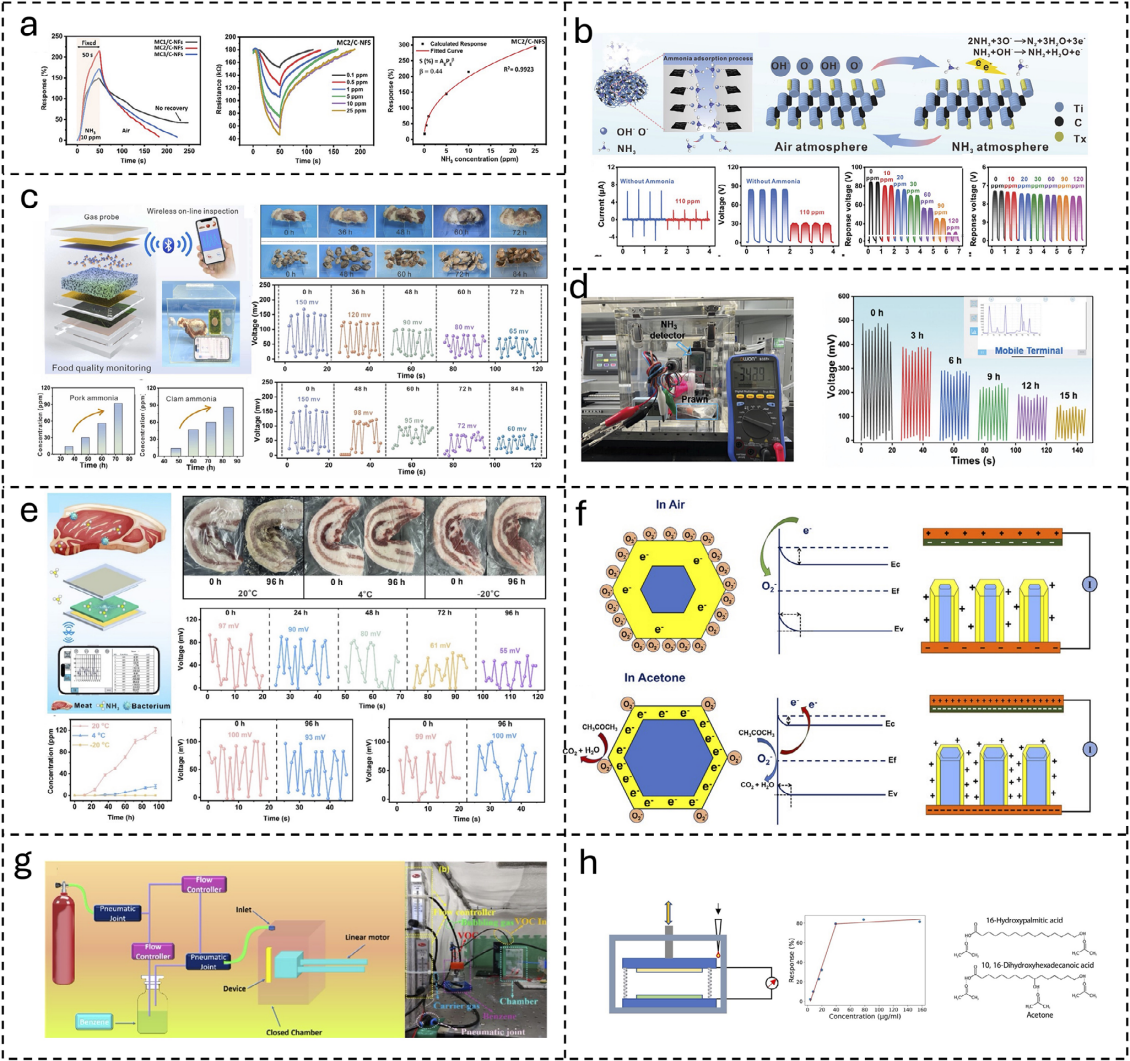
C‐TENG‐based gas sensing systems. (a) Durable MXene/CeO_2_/Cellulose Nanofibers for Self‐Powered Monitoring of Ammonia. Reused from ref. [170]. Copyright 2025, Wiley. (b) Cellulosic triboelectric materials for ammonia sensing. Reused with permission from ref. [171], Under CC BY 4.0. (c) The application of CPC‐aerogel triboelectric gas sensor in food quality monitoring by sensing ammonia. Reused from ref. [172]. Copyright 2024, Elsevier. (d) Using a CNF‐based tribolayer for food quality monitoring by sensing ammonia. Reused from ref. [173] under CC BY 4.0. (e) NH_3_‐sensitive balsa scaffold/PANI/SnO_2_ triboelectric materials for food quality monitoring by sensing ammonia. Reused from ref. [174]. Copyright 2025, Elsevier. (f) ZnO rod‐covered cotton fabric for acetone sensing. Reused from ref. [175]. Copyright 2025, Wiley. (g) TENG with a material pair of cellulose acetate and PVDF/TiO_2_ for acetone sensing. Reused from ref. [176]. Copyright 2019, Elsevier. (h) TENG based acetone sensor utilizing natural plant skin as triboelectric layers. Reused from ref. [177] under CC BY 4.0.

Acetone is another gas molecule that has been detected using C‐TENGs in recent years. A ZnO rod covered cotton fabric has been used as a triboelectric layer for sensing acetone (Figure 9f), and it has a sensitivity of 15% signal change per ppm [175]. The mechanism behind this is the reaction of acetone with the absorbed oxygen species on the ZnO that releases an electron back to the ZnO. In another report (Figure 9g), a TENG with a material pair of cellulose acetate and PVDF/TiO_2_ has also been found to have the capability to sense acetone [176], where TiO_2_ plays a similar role to ZnO. Although these works have demonstrated success in sensing acetone, the main function for sensing is not based on the cellulose materials. Zhang et al. (Figure 9h) have reported a type of TENG‐based acetone sensor utilizing natural plant skin [177]. The mechanism behind this work is the formation of hydrogen bonds between acetone and the rich ─OH groups of cellulose and the surface wax on the plant skin. A detection limit of 3.92 ppm has been achieved, along with linear response ranges of 3.92–39.2 and 39.2–160 ppm.

#### 5.3.7 CO_2_ Capture

CO_2_ capture is of great importance in addressing climate change because CO_2_ is the primary long‐lived heat‐trapping gas driving global warming. Therefore, the application of C‐TENGs as a self‐powered platform for CO_2_ capture and degradation would have great potential. A contact‐electro‐catalytic CO_2_ reduction reaction driven by a TENG composed of CNF and Cu‐PCN/CuO@PVDF has been recently investigated [178]. The Cu atoms on Cu‐PCN enrich electrons during triboelectrification and facilitate electron transfer with absorbed CO_2_ on CNF, which results in the reduction of CO_2_. This method achieves a CO Faradaic efficiency of 96.24%, and a CO yield of 33 µmol/g/h. In another report [179] that employs the same construction to reduce CO_2_ to C_2_H_4_, the triboelectrification between the two surfaces generated a high potential of 400 V, and contact electrons facilitated the reduction of CO_2_. The CNF promotes water dissociation under high humidity, providing protons, while the PVDF provides electrons to complete the contact electrocatalysis. This method achieves a Faradaic efficiency of 63.5%, and the reduction is stable for 60 h of testing. Amine‐functionalized cigarette filters (plasticized cellulose acetate) have been found to increase the adsorption of CO_2_ because the amine sites attract CO_2_, resulting in an adsorption capacity of 1.93 mmol/g, which is 9.2 times higher than that of pristine cigarette filters [180].

#### 5.3.8 Humidity Sensing

Cellulose and its derivatives are rich in ─OH and ─COOH groups, which serve as effective binding sites for moisture, potentially influencing the response of C‐TENGs and making them natural humidity sensors. Table 9 summarizes recent reports on humidity sensing using C‐TENGs. One thing to note is that the definition of detection sensitivity varies across reports, so one needs to be careful when comparing sensitivities.

**TABLE 9:** C‐TENG‐based humidity sensors.

Cellulose material	Counter material	Signal	Linear range	Sensitivity	Refs.
CNF	Ag	Current	65%–85%	−1.5%/RH	[25]
Cellulose/Ti_3_C_2_T_x_	FEP	Voltage	40%–90%	0.8/1% RH	[181]
Rice paper	PET	Voltage	0%–100%	97% (Δ*R*/*R* ^0^)	[182]
Enzymatic paper	PVDF	Voltage	25%–95%	*V*_25%_ /*V*_95% _= 11.3	[183]
Cellulose paper	PI	Voltage	11%–90%		[184]
CNF aerogel	PDMS	Voltage	30%–90%		[163]
Cellulose acetate	MXene	Voltage	44%–88%		[185]
MCC/PEI/FeCl_3_	PTFE	Voltage	20%–80% 80%–98%	0.49 V/%RH −0.38 V/%RH	[186]
Cellulose fiber paper	PVDF/Ag NW	Voltage	30%–90%		[187]
CNF‐BP‐PA/Ag NWs	PVC	Voltage	39%–72%	1.436 V/%RH	[188]
Leek out skin	Leek inner skin	Voltage	28%–56%		[177]

In most studies, the signal used for humidity sensing is the open‐circuit voltage, except for one report that uses a current signal [25]. Such a C‐TENG is made of CNF and Ag as a material pair and shows a linear response range between RH 56% and RH 85% and a sensitivity of −1.5%/RH. For the other humidity sensors, the linear range varies with the material pair and is generally between RH 20% and RH 90%. One thing to note is that the definition of the detection sensitivity of C‐TENG sensors differs across reports, making it difficult to compare sensitivities across reports.

#### 5.3.9 Food Quality Sensing

Smart packaging, where sensors are attached or embedded in the package, has shown its great value in modern transportation and storage. One of the applications is to monitor the freshness of food. Such an application can be based on the response of the C‐TENGs to the gas produced by food, such as seafood, due to the replication of microorganisms. For example, a ZnO nanoparticle‐modified cellulose composite constituting AZP‐TENG has demonstrated the ability to sense ammonia produced by meat [189]. After 16 h, when the level of total volatile basic nitrogen (TVB‐N) reached 15.70 mg/100 g of meat, the measured voltage signal from the AZP‐TENG dropped from 38.8 to 20.32 V. The recovery time of the sensor after removal from the sample is 30 s. To monitor food quality in real‐time, a C‐TENG‐based system, namely TWGSS, has been developed, which includes a wireless transmitter that sends real‐time data on the ammonia level [190]. Similarly, a PFOTES‐Ti_3_C_2_T*_x_*‐CNF film‐based C‐TENG performs the same function of sensing food quality by detecting ammonia levels through its interaction with the surface functional groups of Ti_3_C_2_T*_x,_* such as ─O and ─OH [171]. These examples mostly sense ammonia from meat, which is, however, not the limit of C‐TENGs. A TCNT/PVA/CNT film‐based TENG (CPC‐TENG) has been developed to sense ammonia from both meat and seafood [172].

Although ammonia levels have been widely used as an indicator of food quality, other parameters can also be considered, such as humidity. An enzymatic paper TENG can detect strawberry spoilage within a sealed package. As the water evaporates from the strawberries, the humidity inside the package increases, which changes the TENG's output, resulting in a decrease in voltage and current from 92.11 V and 4.87 µA to 69.36 V and 2.3 µA, respectively [183].

#### 5.3.10 Smart Transportation

Transportation has become increasingly important in modern society, as both passengers and goods need to be moved around. Smart transportation has been a focus of the IoT since the locations of vehicles, driver status, and the condition of goods need to be sensed and communicated via networks. Table 10 summarizes some of the examples of C‐TENGs for smart transportation.

**TABLE 10:** C‐TENGs for smart transportation applications.

Cellulose material	Counter material	Singal	Target	Installed place	Activities	Refs.
Kraft paper	PU	Voltage	Driver	Safety belt	Breathe Forward position	[191]
PPC	PTFE	Voltage	Driver	Bracelet	Steering wheel Engage gear Push button	[192]
MCC/CS	SA	Voltage	Kiwi	Packaging box	Collisions	[193]
Kraft paper	PTFE	Voltage	Packaging box	Conveyor belt	Position	[194]

A kraft paper‐based TENG attached to the belt can be used to monitor the driver's forward position and turning actions (Figure 10a) [191]. The TENG is highly sensitive to the driver's normal and deep breathing, generating distinct triboelectric signals for analysis. Moreover, it can capture the driver's forward position, which is important for safety issues while driving. In another report (Figure 10b), a driver has worn a bracelet made of a polyvinyl alcohol‐polyacrylamide double network hydrogel in the presence of sodium alginate and tannic acid‐modified cellulose nanocrystals (PPC) and PTFE as TENG components to sense steering wheel rotation, push buttons, and gear engagement [192].

**FIGURE 10:**
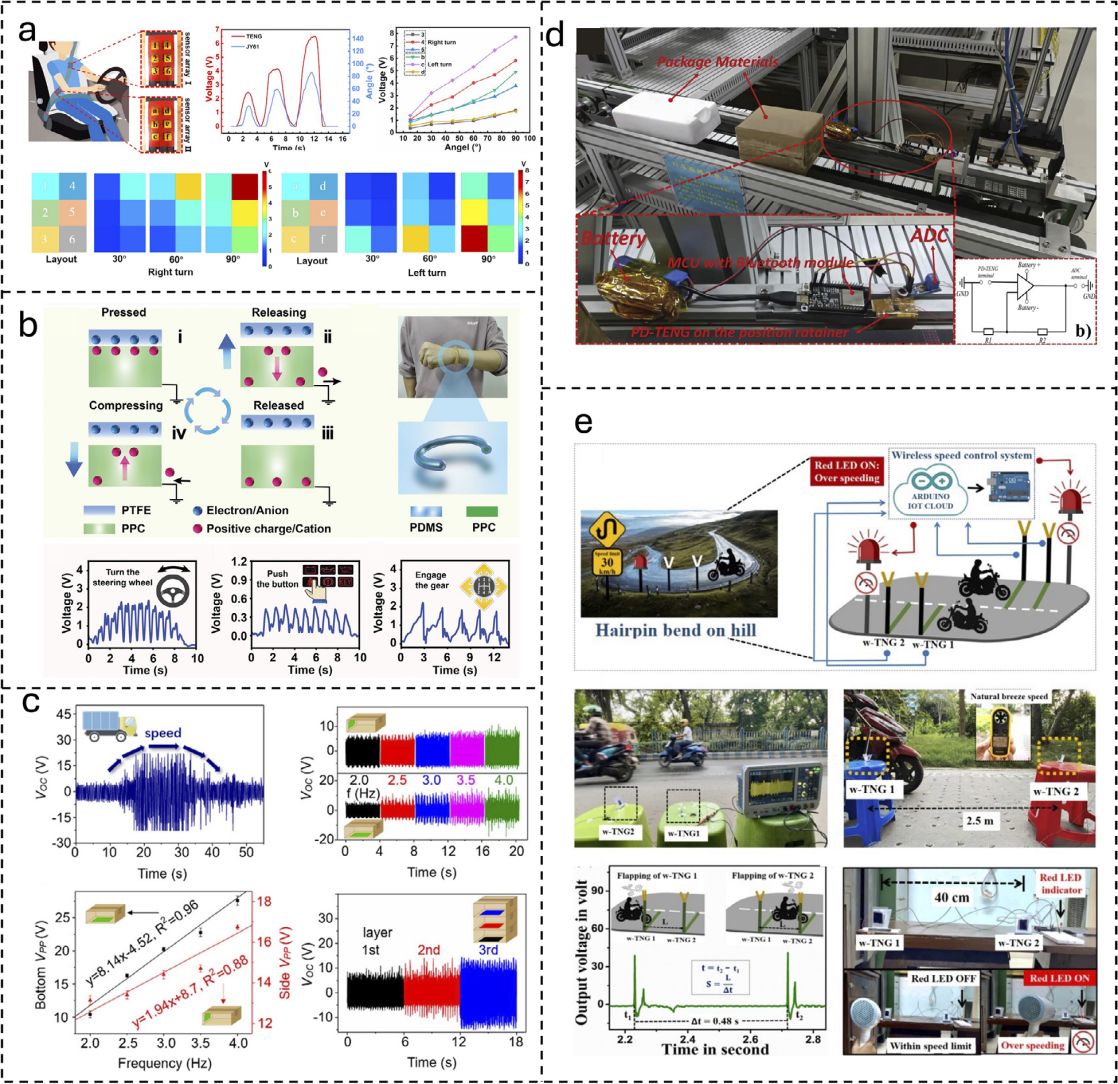
C‐TENGs for smart transportation. (a) A kraft paper‐based TENG attached to the belt to monitor the forward position and turning actions of the driver. Reused from ref. [191] with permission. Copyright 2019, Elsevier. (b) Sodium alginate and tannic acid‐modified cellulose nanocrystals (PPC) and PTFE as TENG components to sense steering wheel rotation. Reused from ref. [192] with permission. Copyright 2023, Wiley. (c) A microcrystalline cellulose/chitosan (MCC/CS) composite and sodium alginate (SA) constructed TENG sensor for monitoring kiwi collision inside a box. Reused from ref. [193] with permission. Copyright 2024, ACS. (d) Kraft paper based TENG for monitoring the position of boxes. Reused from ref. [194] with permission. Copyright 2022, Elsevier. (e) A nylon layer cast onto a microfibrous cellulose paper for use in a TENG sensor for speed monitoring. Reused from ref. [195] with permission. Copyright 2025, Elsevier.

Besides monitoring drivers’ activities, other issues have been studied recently. For example, the collision of kiwi fruits inside a box can be detected using a TENG constructed from a microcrystalline cellulose/chitosan composite and sodium alginate (SA) [193]. The vibration of the kiwi inside the box generates a voltage signal that is linearly related to the vibrational frequency (Figure 10c). Moreover, the layer of the box inside the car shows different signal intensities, suggesting the possibility of identifying the signal's location. Based on the triboelectrification between kraft paper and PTFE (Figure 10d), a series of free‐standing mode TENGs have been installed on a conveyor belt to monitor the position of a box [194]. By analyzing the signal order from a series of TENGs, the position of the box can be sensed and communicated wirelessly.

Cellulose materials can also be used as frames or supporting layers for TENG sensors in smart transportation applications. A nylon layer cast onto a microfibrous cellulose paper can serve as a triboelectric positive layer in a TENG sensor for speed monitoring [195]. The triboelectric signal from the TENG is generated by wind from passing cars, and it can be translated into an alert if over‐speeding (Figure 10e). In another study, honeycomb holes cut from paperboard are used as a frame for a desiccant‐based TENG, in which desiccant balls bounce between two PTFE films mounted at the open ends of the honeycomb holes [197]. The signals from the sensor can be used to analyze the temperature and humidity of packaged goods during transportation.

## 6 Challenges

### 6.1 Moisture Management and Long‐Term Stability

Hygroscopicity remains the major challenge for cellulose‐based TENGs, as the absorbed water can significantly reduce their performance. Reducing water uptake in the materials by creating multilayer barriers that combine organic hydrophobic molecules (silanes, fluoropolymers) or ultrathin inorganic layers is a promising strategy for future studies.

### 6.2 Mechanochemical Durability and Wear

Repeated contact‐separation during the operation of TENGs results in fibrillation, surface abrasion, and a gradual loss of the microstructure of the cellulose films, necessitating extra care to enhance durability. Cross‐linking and nanocellulose reinforcement may be useful methods to extend the lifetimes of the triboelectric.

### 6.3 Theoretical Understanding

Charge transfer pathways in hydrogen‐bonded, polar interfaces for cellulose materials are still not fully understood. Kelvin probe force microscopy under controlled humidity, combined with multiscale simulation, can provide more information for fundamental understanding of the charge transfer on cellulose materials. Quantitative models that include molecular dipoles, trap distributions, and macroscopic charge density are highly demanded for future studies.

### 6.4 Life‐Cycle Considerations

Although cellulose materials are normally environmentally friendly, environmental analysis for C‐TENGs is still needed to understand the material and energy flow during the whole life cycle. Life‐cycle assessment (LCA) analysis tailored to C‐TENGs should be performed and include metrics for embodied energy, greenhouse gas emissions, and recyclability of electrodes and encapsulants.

### 6.5 Engineering Cellulose Triboelectric Materials

The transition of C‐TENGs from laboratory studies to viable, sustainable power sources and sensing devices relies on the development of large‐scale production. While many reports focus on novel chemistries, the practical challenge lies in producing these materials at a cost, speed, and volume that can compete with incumbent fluoropolymers.

#### 6.5.1 Scalable Synthesis of Triboelectric Cellulose Platforms

Before device fabrication, the active triboelectric material must be produced in a high‐throughput, reproducible manner.

- Regenerated cellulose films: While lab‐scale solvent casting is commonly used to produce regenerated cellulose films, industrial‐scale production typically requires continuous processes. Methods such as slot‐die coating of cellulose solutions onto a carrier, followed by drying, could be used. However, the engineering challenge here is controlling the solvent removal rate and regeneration conditions to ensure uniform thickness, desired crystallinity (Cellulose II), and optimal surface properties, as these directly govern triboelectric performance.
- Nanocellulose production and film formation: For nanocellulose (CNCs, CNFs, and BCs), the engineering challenge has two aspects. First is the industrial‐scale extraction (e.g., large‐scale hydrolysis for CNCs or high‐pressure homogenization for CNFs). Second is the formation of large‐area films, which is often accomplished via continuous filtration or papermaking‐style roll‐to‐roll processes that dewater the nanocellulose slurry to form a cohesive, paper‐like film.

#### 6.5.2 High‐Throughput Device Fabrication and Integration

A key advantage of cellulose is its compatibility with existing high‐throughput technologies from the paper and flexible electronics industries.

##### 6.5.2.1 Roll‐to‐Roll (R2R) Processing

This is the ultimate goal for low‐cost, large‐area production. As demonstrated by Zhang et al. [19], R2R coating machines can be used to deposit functional layers, such as nano‐graphite electrodes, onto a paper substrate at high speeds (e.g., 25 m/min). This approach can be extended to deposit the active triboelectric layer, electrode, and even a passivation layer in a continuous sequence, creating application‐ready modules.

##### 6.5.2.2 Printing Technologies

Cellulose substrates are ideal for various printing methods. However, industrial printing technologies are not widely used for making C‐TENG components, including:

- Screen printing: Widely used for depositing thick‐film electrodes (e.g., silver paste, carbon/graphite) and dielectric layers. Its compatibility with paper is well‐established.
- Inkjet printing: Offers high‐resolution patterning, suitable for depositing nanocellulose‐based inks or conductive inks, enabling the creation of complex electrode geometries for sensors and integrated circuits.
- Slot‐die and doctor‐blading: These methods are R2R‐compatible and ideal for laying down uniform, large‐area films of regenerated cellulose solutions or nanocellulose suspensions.

Figure 11 summarizes the challenges in research and the lab‐ and engineering‐scale production of C‐TENGs. To put the C‐TENGs into a commercial product, there is still a long way to go. The performance of the C‐TENGs still needs to be improved to compete with engineering polymers. Market analysis and development are other important issues to be addressed before the commercialization of C‐TENGs.

**FIGURE 11:**
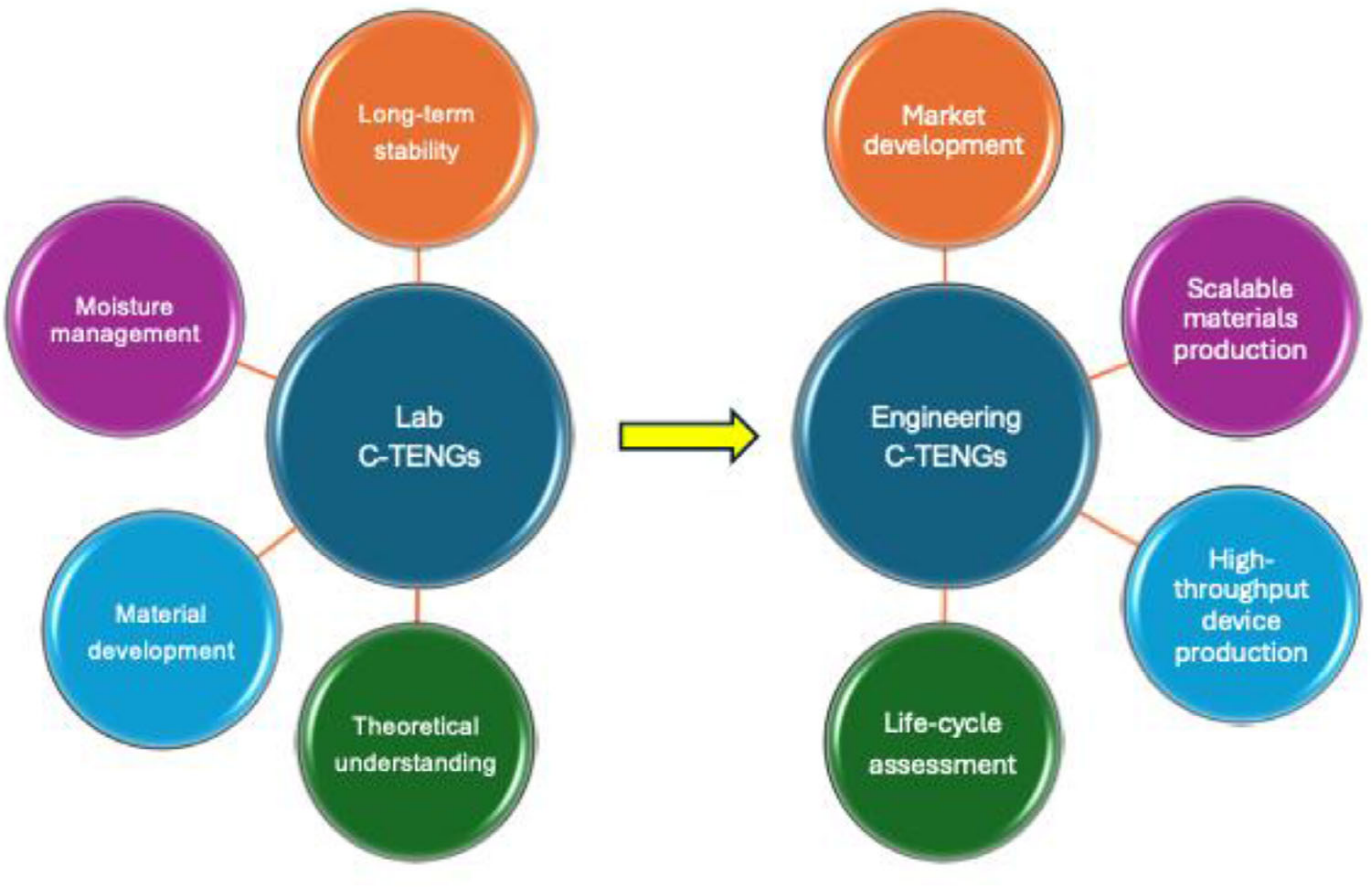
The challenges for C‐TENGs from lab to engineering.

## 7 Conclusions

Cellulose is transitioning from a promising sustainable dielectric to an engineered platform for scalable energy harvesting. Advances in solvent engineering, surface chemistry, and hybrid nanocomposites have unlocked performance previously associated primarily with fluoropolymers. The immediate priorities are (i) humidity‐tolerant surface chemistries that preserve charge, (ii) designing cellulose triboelectric materials with the aim of industrial‐scale production, which could replace the engineering polymer at a larger scale. In parallel, community standards for measurement and reporting will reduce ambiguity, allowing for meaningful comparisons across studies and applications. With these elements in place, cellulose‐based TENGs can form a new class of green, self‐powered systems that span wearables, smart infrastructure, and distributed environments.

## Author Contributions

**Renyun Zhang**: conceptualization, data curation, formal analysis, visualization, writing – original draft, writing – review editing. **Dabo Chen**: writing – original draft, writing – review editing, resources. **Jiayong Li**: writing – review editing, writing – original draft. **Peihong Wang**: writing – original draft, writing – review editing, resources.

## Conflicts of Interest

The authors declare no conflicts of interest.

## Data Availability

The data that support the findings of this study are available on request from the corresponding author. The data are not publicly available due to privacy or ethical restrictions.
